# Anti-trypanosomal quinazolines targeting lysyl-tRNA synthetases show partial efficacy in a mouse model of acute Chagas disease

**DOI:** 10.1126/scitranslmed.adu4564

**Published:** 2025-07-09

**Authors:** Lindsay B. Tulloch, Hugh Tawell, Annie E Taylor, Marta Lopes Lima, Alice Dawson, Sandra Carvalho, Richard J. Wall, Victoriano Corpas-Lopez, Gourav Dey, Jack Duggan, Luma Godoy Magalhaes, Leah S. Torrie, Laura Frame, David Robinson, Stephen Patterson, Michele Tinti, George W Weaver, William J Robinson, Monica Cal, Marcel Kaiser, Pascal Mäser, Peter Sjö, Benjamin Perry, John M. Kelly, Amanda Fortes Francisco, Avninder S. Bhambra, Susan Wyllie

**Affiliations:** 1 Wellcome Centre for Anti-Infectives Research, School of Life Sciences, University of Dundee, Dundee, UK; 2 Department of Chemistry, Loughborough University, Loughborough, LE11 3TU, UK; 3 Leicester School of Allied Health Sciences, De Montfort University, The Gateway, Leicester, LE1 9BH, UK; 4 Drug Discovery Unit, School of Life Sciences, University of Dundee, Dundee, UK; 5 Swiss Tropical and Public Health Institute, 4123 Allschwil, Switzerland; 6 University of Basel, 4003 Basel, Switzerland; 7 Drugs for Neglected Diseases initiative (DNDi), 1202 Geneva, Switzerland; 8 London School of Hygiene and Tropical Medicine, London, UK

## Abstract

The protozoan parasite *Trypanosoma cruzi* causes Chagas disease, which is among the deadliest parasitic infections in Latin America. Current therapies are toxic and lack efficacy against chronic stage infection thus new drugs are urgently required. Here, we describe a new series of quinazoline compounds with potential against *T. cruzi* and the related trypanosomatids *T. brucei* and *Leishmania donovani in vitro*. We demonstrate partial efficacy of a lead quinazoline compound in a mouse model of acute Chagas disease. We conducted mechanism of action studies using several orthogonal approaches and confirmed that this new quinazoline compound series targeted the ATP-binding pocket of lysyl-tRNA synthetase 1 (KRS1) in *T. cruzi*. A high-resolution crystal structure of KRS1 bound to the drug indicated binding interactions that led to KRS1 inhibition. Our study identifies KRS1 as a druggable target for treating *T. cruzi* infection. This quinazoline series shows potential for treating Chagas disease but will require further development to become a future treatment for this neglected disease.

## Introduction

Chagas disease, caused by infection with the protozoan parasite *Trypanosoma cruzi*, is responsible for more fatalities than any other parasitic disease in Latin America and is a leading cause of infectious cardiomyopathy worldwide (1). Chagas disease cases in the USA and Europe are on the rise, driven predominantly by migration from endemic countries (2, 3). However, it should be noted that global warming is driving a shift in the geographical range of the Chagas disease vector, bugs of the Reduviidae family, raising the possibility that an increasing number of countries may experience endemic transmission (4). An estimated 6-7 million people are infected with *T. cruzi* and a further 75 million people are at risk of infection (www.who.int) (3). Approximately 30% of infected individuals progress to chronic, symptomatic disease that commonly affects the cardiovascular and digestive systems causing cardiomyopathy or digestive tract megasyndromes (5). Both forms of the disease are associated with major morbidity and mortality. At present, only two drugs, benznidazole and nifurtimox, are approved for the treatment of Chagas disease. Both are nitroheterocyclic prodrugs that require reduction catalyzed by a type I nitroreductase (NTR1) for biological activity (6–8). These drugs are associated with toxic side-effects that are often so severe that they prevent patients from completing treatment regimens (9, 10). In addition, both drugs are less efficacious in the chronic stage of the disease, the stage in which the vast majority of patients are diagnosed. Thus, new therapeutics with improved efficacy and reduced toxicity are urgently required.

Anti-chagasic drug discovery has long been hampered by a lack of dedicated resources. The most recent clinical trials have focused on assessing drugs repurposed from other disease indications rather than new chemical entities optimized for Chagas disease. To date, this strategy has proven ineffective. Most notably, the anti-fungal drug posaconazole that targets C14α sterol demethylase (CYP51), a key enzyme in the sterol biosynthetic pathway, failed in phase II clinical trials, with the vast majority of patients relapsing within a year of treatment (11). This was despite preliminary data indicating promising *in vitro* potency and *in vivo* efficacy in murine models of infection (12, 13). Similarly, the nitroimidazole fexinidazole, an oral drug currently used in the treatment of human African trypanosomiasis (14), proved ineffective in phase II proof-of-concept trials for Chagas disease. The clinical failure of both drugs has been partially attributed to their inability to eradicate persister forms of *T. cruzi* with reduced cell proliferation rates (15–17). Collectively, these studies emphasize the need for dedicated Chagas disease drug discovery programs aimed at delivering compounds capable of killing all developmental forms of this parasite at all stages of disease.

Here, we describe our orthogonal genetic, molecular, and biochemical studies to determine the molecular target of a quinazoline series of compounds demonstrating promising anti-chagasic activity. Screening of this series against the intracellular amastigote form of *T. cruzi*, identified hits that demonstrated *in vitro* potency superior to the current standard of care benznidazole and with limited mammalian cell cytotoxicity. Two compounds (DMU371 and DMU759) from this series were subjected to comprehensive drug target deconvolution studies leading to the identification of *T. cruzi* lysyl-tRNA synthetase (*Tc*KRS1) as the molecular target of both quinazolines. Most notably, proof-of-concept studies in a murine model of acute Chagas disease demonstrated that DMU579 was capable of reducing parasitemia by up to 81%. Collectively, these studies identified KRS1 as a chemically validated drug target for the treatment of *T. cruzi* infection.

## Results

### Quinazoline compounds are potent inhibitors of kinetoplastid parasites

**Fig. 1:**
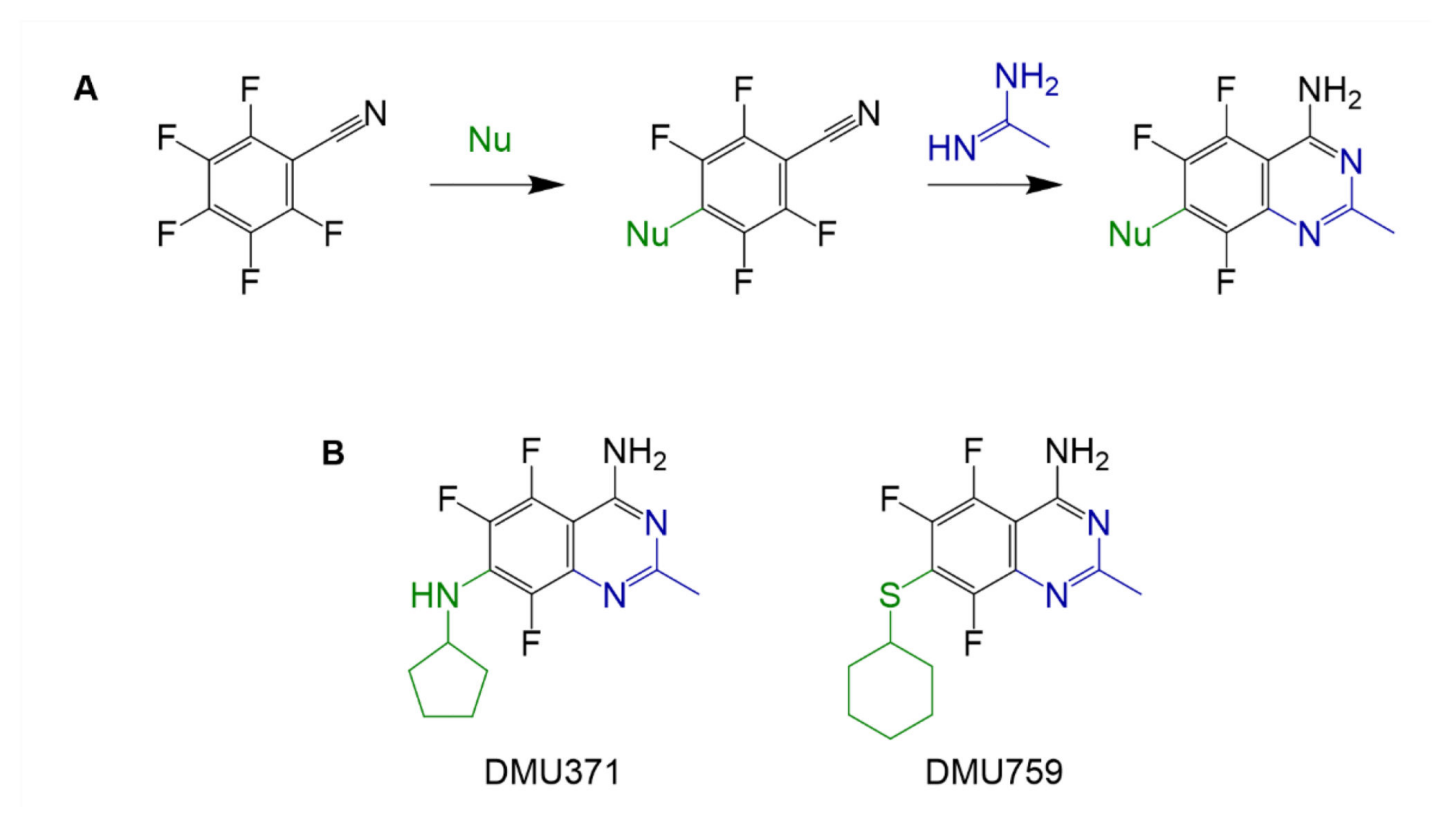
Generation of trifluorinated quinazolines. (**A**) Schematic shows the synthesis strategy for generating trifluorinated quinazolines. (**B**) Shown are the two lead compounds, DMU371 and DMU759, that were further characterized and their mechanism of action investigated. Nu denotes nucleophilic addition.

Perfluorinated building blocks can be exploited to rapidly generate a broad range of polyfluorinated drug-like molecules. Previous studies from our group used this strategy to generate a series of 2,6-disubstituted-4,5,7-trifluorobenzothiophenes that demonstrated promising activity against *T. cruzi*, as well as the related kinetoplastid parasites *T. brucei spp*., causative agents of human and animal African trypanosomiasis, and *Leishmania donovani*, responsible for visceral leishmaniasis (18). In the course of these studies, it became evident that this fused 6,5 heterocyclic core skewed activity specifically towards *T. brucei spp*., rather than *T. cruzi* and *L. donovani*. Given that the initial goal of our current study was to develop compounds with broad spectrum anti-kinetoplastid activity, we employed a scaffold hopping approach to design fused heterocyclic compounds with the trifluorinated core maintained that proved essential for all phenotypic activity. Ultimately, reactions of pentafluorophenylacetonitrile with ring-forming reagents, including amidines, were performed to yield new drug-like trifluorinated quinazolines (Fig. 1A).

Early compounds developed within this series demonstrated *in vitro* potency against a range of kinetoplastid parasites indicating the broad utility of the fluorinated quinazoline scaffold. A combinatorial chemistry approach was used to further explore the impact of modifications at positions 2, 4 and 7 of this scaffold. Mouse liver microsome assays revealed that compounds within this series with demonstrated potent anti-kinetoplastid activity (EC_50_ <1 μM) were prone to high rates of intrinsic clearance indicative of metabolic instability. However, this liability was not linked to the quinazoline core itself. Therefore, two lead compounds, DMU371 and DMU759 (Fig. 1B) were selected for further study. These two compounds demonstrated efficient anti-kinetoplastid activity (Table 1 and table S1) and acceptable ADME profiles (excluding metabolic stability) (table S2). Thus, DMU371 and DMU759 were selected for subsequent target deconvolution and validation studies.

Understanding the mechanism of action and molecular targets of compounds identified through phenotypic screening can greatly facilitate the drug discovery process. Once the molecular target is known, target-focused and structure-guided strategies to improve compound potency, selectivity and pharmacokinetic properties, such as metabolic stability, can be developed. Knowing the target of a phenotypically active compound can be vital in efficiently ceasing development of compounds acting through an undesirable mechanism of action. This is particularly relevant to drug discovery for Chagas disease where a considerable proportion of hits identified through phenotypic screening have been found to target CYP51 (15), a discredited target following the failure of posaconazole in clinical trials (11). With this in mind, our primary goal was to determine the molecular targets of this quinazoline series to support further development.

### Screening of quinazolines against a genome-wide overexpression library

**Fig. 2:**
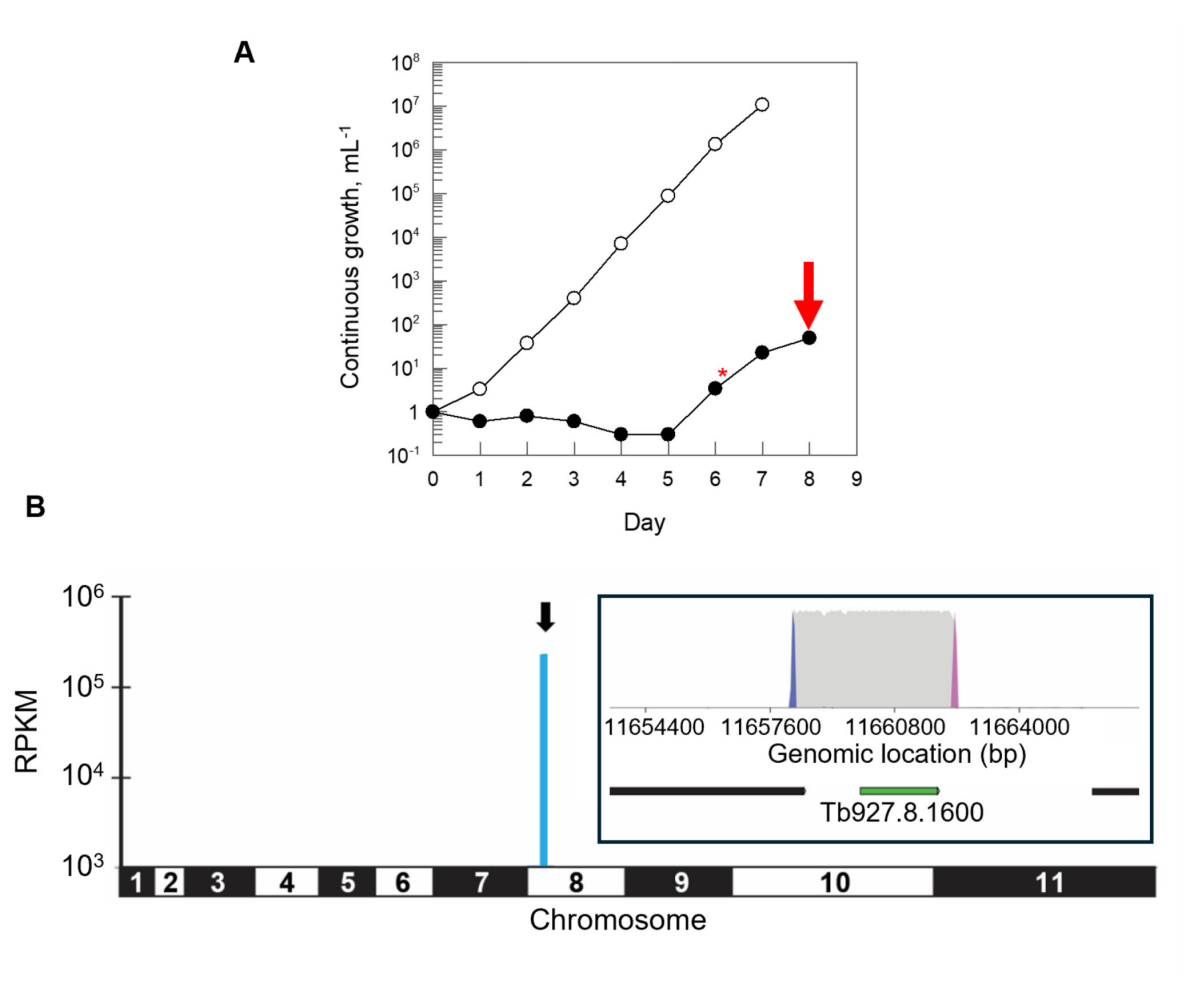
Genome-wide overexpression library screen with DMU371. The library comprises a pooled population of bloodstream form T. brucei transfected with overexpression plasmids containing genomic DNA fragments between 3 and 10 kb in size. The final transfected library provides 10-fold genome coverage with >95% of T. brucei genes represented. The parasite library is grown in the presence of test drug and genomic DNA is harvested from those able to survive selection. Next generation sequencing identifies the genomic fragments harbored by “drug resistant” parasites revealing overexpressed proteins and potential drug targets (19). (**A**) Growth of the genome-wide overexpression library in the presence or absence of DMU371 selection at a concentration equivalent to twice the established EC_50_ value. Asterisk indicates sub-culturing of the culture and addition of fresh compound. The arrow indicates when the library was harvested for genomic DNA preparation. (**B**) Shown is the genomewide map resulting from the overexpression library screen with DMU371. RPKM, reads per kilobase of transcript per million mapped reads. Inset shows the top fragment hit of this library screen containing the full coding sequence of lysyl-tRNA synthetase (Tb927.8.1600). Gene of interest is highlighted in green, other protein-coding regions are indicated in black. Blue and pink peaks represent forward and reverse barcodes (in the sense orientation), respectively. Grey peaks are all other reads (table S3).

Taking advantage of the fact that our quinazoline series of compounds demonstrated potent activity against all three kinetoplastid parasites (Table 1 and table S1), we screened DMU371 against our genome-wide overexpression library in *T. brucei* (19). The principle underpinning this gain-of-function screen was that overexpression of a drug target would confer resistance to the corresponding drug by increasing the pool of functional protein or by reducing free drug through binding. The bloodstream form of *T. brucei* was transfected with a pooled population of plasmids containing genomic DNA fragments between 3 and 10 kb in size. The final transfected library provided 10-fold genome coverage with >95% of *T. brucei* genes represented. DMU371 was screened against the library at 1.2 μM, equivalent to twice the established EC_50_ value of the compound against bloodstream trypanosomes (table S1). Growth of the library under selection with DMU371 was suppressed relative to the unselected library during the first 5 days of selection, however, it recovered strongly prior to harvesting on day 8 (Fig. 2A). Following compound selection, plasmids maintained by the “resistant” parasite population were harvested and analysed by next-generation sequencing. Mapping of enriched inserts to the *T. brucei* 927 strain assembled genome revealed that 97% of all mapped reads (4.4 million) aligned to a single region of chromosome 8. This genomic fragment encompassed the entire open reading frame encoding lysyl tRNA synthetase 1 (*KRS1*, Tb927.8.1600), as well as part of the neighboring gene encoding a putative ubiquitin protein ligase (Tb927.8.1590) (Fig. 2B, table S3). As the only intact gene encoded by this enriched genomic fragment, we hypothesized that overexpression of KRS1 likely conferred resistance to DMU371 thus identifying this enzyme as a putative molecular target of this quinazoline.

### Genetic validation of KRS1 as the target of DMU371 and DMU759

Aminoacyl-tRNA synthetases (aaRS), ubiquitous enzymes that are essential for protein translation, are considered attractive therapeutic targets in a number of infectious diseases, including malaria, tuberculosis and cryptosporidiosis (20–23). These enzymes catalyze aminoacylation of transfer RNA (tRNA) with their appropriate amino acids. This process, often referred to as tRNA-charging, occurs in two distinct reaction steps: amino acid activation where the amino acids are activated by ATP to yield AMP-activated amino acids followed by tRNA acylation where cognate amino acids are transferred onto tRNAs (Fig. S1). Subtle structural differences in the active sites of a variety of pathogen versus human aaRS have been successfully exploited to design potent and selective inhibitors of these enzymes (21, 22). *T. brucei*, and kinetoplastids more broadly, maintain two copies of *KRS*, both encoded by the nuclear genome. *Tb*KRS2 more closely aligns to the equivalent aaRS in bacteria, maintaining a *C*-terminal extension responsible for targeting the protein to the mitochondria where it is cleaved to produce a mature, functional protein (Fig. S2) (24). *Tb*KRS1, the putative target of DMU371, is more similar to aaRS from apicomplexan parasites and localizes to the cytoplasm. Across the three major kinetoplastid pathogens, the similarity between KRS1 and KRS2 ranges from 30 to 40% (Fig. S2).

The results of our overexpression library screen suggested that DMU371 was a specific inhibitor of *T. brucei* KRS1. To validate this hypothesis, we next generated a clonal *T. brucei* cell line specifically overexpressing *Tb*KRS1 (*Tb*KRS1^OE^). Elevated expression of *Tb*KRS1 in these transgenic parasites, relative to wildtype parasites, was confirmed by label-free mass spectrometry quantification (Fig. S3A). Compared to wildtype *T. brucei* parasites, *Tb*KRS1^OE^ parasites demonstrated ~5-fold reduction in susceptibility to the established selective KRS1 inhibitor DDD01510706 (20, 25), indicating that overexpression of *Tb*KRS1 was able to functionally complement the compound-inhibited enzyme (table S4). In keeping with our library screen, overexpression of *Tb*KRS1 in bloodstream trypanosomes conferred a similar 7-fold resistance to the representative quinazoline DMU371 (table S1) as well as 9-fold resistance to DMU759. These data supported our hypothesis that KRS1 was the molecular target of DMU371 in *T. brucei* and indicated that compounds within our quinazoline series likely shared the same mechanism of action.

Given that DMU371 and DMU759 were pan-active against all kinetoplastid parasites screened to date (Table 1, table S1), we next sought to determine whether overexpression of KRS1 homologs in *L. donovani* and *T. cruzi* would also confer resistance to both compounds. Transgenic cell lines overexpressing *L. donovani* and *T. cruzi* KRS1 enzymes were generated, with successful overexpression again confirmed by label-free mass spectrometry quantification (Fig. S3). *L. donovani* promastigotes overexpressing *Ld*KRS1 were 13-fold and 4-fold less susceptible to DMU759 and DMU371, respectively (table S1). Similarly, *T. cruzi* epimastigotes overexpressing *Tc*KRS1 (*Tc*KRS1^OE^) demonstrated 10-fold resistance to these compounds compared to wildtype *T. cruzi* epimastigotes (Table 1). Importantly, when grown as intracellular amastigotes within Vero cells, the medically relevant mammalian infective stage of the parasite, *Tc*KRS1^OE^ retained resistance to both compounds (Table 1). Collectively, these data supported KRS1 as the common molecular target of these quinazolines in all three major kinetoplastid pathogens.

### Quinazolines DMU371 and DMU759 bind to the ATP-binding pocket of KRS1

Previous studies have confirmed that DDD01510706, a chromone inhibitor of *P. falciparum* and *Cryptosporidium parvum*, binds to the ATP-binding pocket of KRS1 in both parasites (21, 25). Mutation of a serine residue (S344) to a leucine residue within the ATP-binding pocket of the *P. falciparum* KRS1 conferred resistance to DDD01510706, with the bulkier leucine residue preventing binding of the inhibitor. Notably, the serine residue is conserved in all three kinetoplastid KRS1 enzymes.

To investigate the possibility that DMU371 and DMU759 exploited the same ATP-binding pocket as DDD01510706, *T. brucei, T. cruzi* and *L. donovani* cell lines bearing a serine-to-leucine mutation in the residue equivalent to *Pf*KRS^S344^ were generated, either through oligonucleotide targeting (*Tc, Tb*) (26) or CRISPR-Cas9 gene editing (*Ld*) (27). All three mutated cell lines (LdKRS1^S324L^, *Tc*KRS1^S319L^ and *Tb*KRS1^S323L^) demonstrated reduced susceptibility to both quinazolines (Table 1, table S1) indicating that both compounds likely occupied the ATP-binding pocket of the KRS1 enzyme. Interestingly, these point mutations conferred marked resistance to DMU759 in *L. donovani* (12-fold), *T. cruzi* (41-fold) and *T. brucei* (58-fold), relative to susceptibility of wildtype parasites (Table 1, table S1). Similar resistance was observed when the cell lines were screened with DDD015150706 (table S4). However, these transgenic parasite lines were only moderately resistant (between 3-fold and 5-fold) to DMU371 perhaps suggesting that this compound exploited a slightly different binding position compared to DMU759 and DDD01510706. Our data suggested that both quinazolines targeted the ATP-binding pocket of KRS1 in all three kinetoplastid parasites. We next focused on characterizing these inhibitors further against *T. cruzi*.

### Direct evidence of on-target engagement

The gold-standard in target deconvolution studies is to provide evidence of the compound or drug of interest directly binding to its molecular target. This can be achieved in a variety of ways (reviewed in (28). Here, we aimed to provide unbiased confirmation of DMU371 and DMU759 binding to their molecular target (KRS1) in *T. cruzi* using isothermal proteome profiling (iTPP) (25), a rationalised version of our standard thermal proteome profiling assay (TPP) (29). Both versions of TPP are based on the principle that binding of a drug to its protein target can alter the thermal stability of that target. In the iTPP assay, the relative abundance of proteins in cell lysates is monitored at a single temperature in the presence and absence of test compounds. Increased abundance of a specific protein in the presence of a compound is indicative of thermo-stabilisation of the target as a result of a direct interaction with the ligand.

**Fig. 3:**
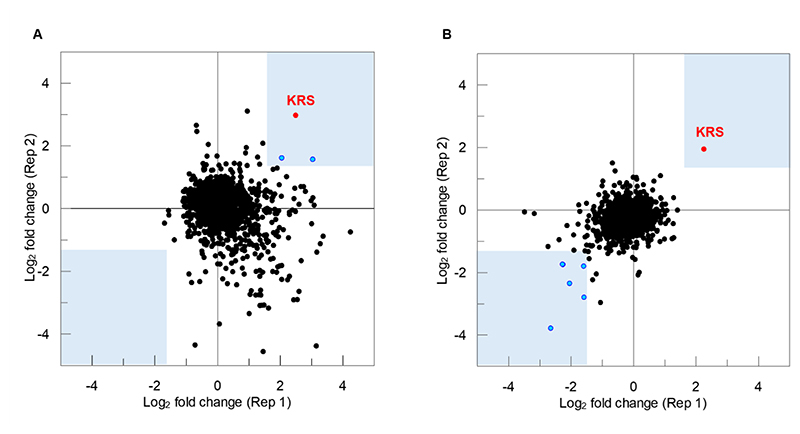
Isothermal TPP for proteome scale identification of quinazoline targets. Isothermal TPP plots show log_2_ fold change in abundance between compound-treated and untreated T. cruzi epimastigote lysates subjected to thermal shock at 48°C across two biological replicates. All proteins identified with >2 unique peptides are shown. “Hits” demonstrating a >1.5-fold shift in abundance (increase or decrease) are shown in blue. TcKRS1 is indicated in red. Hits for experiments with DMU371 and DMU759 are noted in tables S5 and S6, respectively.

In the current study, whole cell lysates of *T. cruzi* epimastigotes were prepared and exposed to DMU371 or DMU759 at 10 times their respective EC_50_ values or DMSO as a vehicle control for 30 minutes. Our previous studies illustrated that the *T. cruzi* epimastigote proteome has a T_m_ (temperature at which 50% of all proteins are denatured) of ~46°C (29). To maximise the chances of identifying proteins stabilized in the presence of compound, treated and control lysates were incubated at 48°C. Following incubation, insoluble (denatured) proteins were removed, and the abundance of remaining soluble proteins in each treated and control sample was determined by quantitative mass spectrometry. Proteins demonstrating a >1.5-fold shift in abundance (increase or decrease) in the presence of drug were considered as “hits” and putative targets of the compound. *Tc*KRS1 was identified as the top hit and most compelling candidate target of DMU371 and DMU759, with the relative abundance of this enzyme increasing considerably in the presence of both compounds and across two biological replicates (Fig. 3; tables S5, S6). In addition to *Tc*KRS1, two proteins were modestly but consistently stabilized in the presence of DMU371 (table S5), whereas five proteins were identified as hits destabilized in the presence of DMU759 (table S6). These data provided further evidence that *Tc*KRS1 was the primary target of these quinazoline compounds.

### Inhibition of *Tc*KRS1 enzyme activity

KRS1-mediated synthesis of lysyl tRNA occurs in two distinct steps. In the first step, lysine and ATP are combined to form a lysyl-5′-AMP intermediate with the concomitant release of pyrophosphate. In the second step of the reaction, the lysyl-5′-AMP intermediate is conjugated to the 3′ of the cognate tRNA. To confirm that binding of our quinazoline compounds inhibited enzyme activity, *Tc*KRS1 was recombinantly expressed and purified. The enzymatic activity of the recombinant protein was monitored based on a previously reported biochemical assay (25). The assay monitors the first step of the aminoacylation reaction and specifically the production of pyrophosphate that is converted to free phosphate by a coupled enzyme (pyrophosphatase), the free phosphate is then quantified by the addition of the reagent BIOMOL Green (Fig. S1). As expected, both DMU371 and DMU759 proved to be potent inhibitors of *Tc*KRS1 activity in this assay with mean IC_50_ values of 185 nM (95% confidence interval, 98–352 nM, *n*=7) and 15 nM (95% confidence interval, 5–43 nM, *n*=4), respectively. Importantly, in parallel assays, these quinazolines were not potent inhibitors of the human ortholog of KRS1, with mean IC_50_ values of 7170 nM (95% confidence interval, 2860–18010 nM; *n*=4) and 9800 nM (95% confidence interval, 5240–18300 nM; *n*=4) for DMU759 and DMU371, respectively (Table 2). In addition, only partial inhibition of the human KRS1 enzyme was observed, in contrast to *Tc*KRS1, where full inhibition of the enzyme was measured. These IC_50_ values were consistent with the degree of selective toxicity demonstrated by both compounds against *T. cruzi* parasites (epimastigotes and intracellular amastigotes) relative to a human HepG2 cell line (Table 1).

### Crystal structures reveal key inhibitor binding interactions in the KRS ATP-binding pocket

**Fig. 4:**
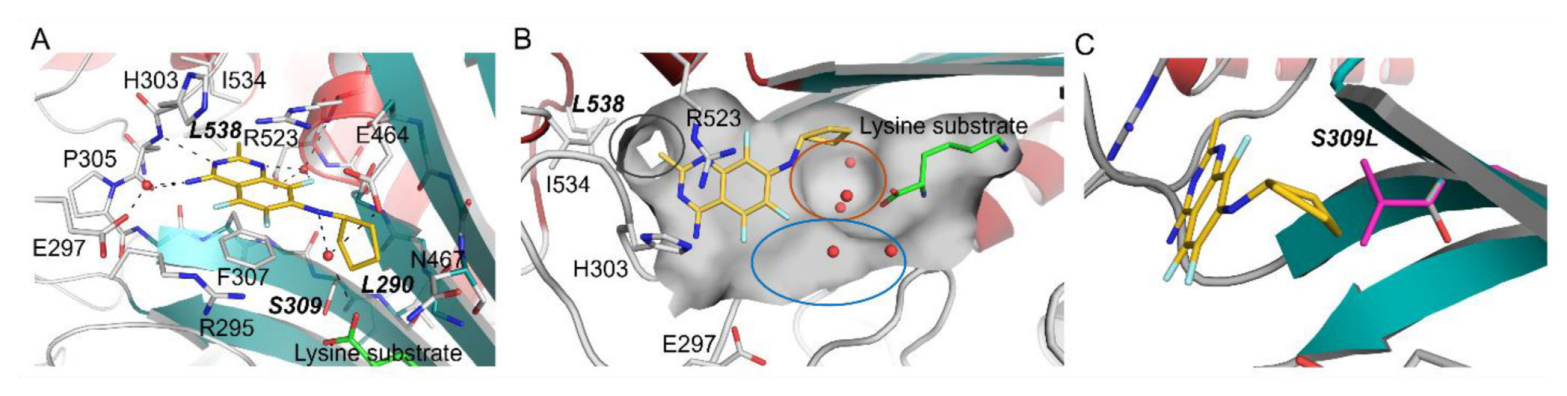
Crystal structure of TcCpKRS1 with DMU371 bound to the active site. (**A**) Shown is the crystal structure of TcCpKRS1 with DMU371 (yellow) bound to the active site in the presence of lysine substrate (green). Water molecules are indicated in red and hydrogen bonding interactions formed with water molecules are represented as black dashed lines. Key residues are labeled using CpKRS1 numbering. C. parvum residues mutated to their T. cruzi counterparts are shown in bold italics. (**B**) Three unoccupied regions of the active site that could be exploited for future quinazoline analog development are highlighted with red, blue and dark grey ovals. The surface of the enzyme is shown in grey and water molecules are shown in red. (**C**) The most likely conformation of S309L is shown in magenta. Proximity to the cyclopentyl moiety of DMU371 (yellow) and potential for steric clash is evident.

To gain insight into the molecular interactions that these inhibitors made within the ATP-binding pocket of KRS1, we aimed to generate a high-resolution crystal structure with ligand bound. In the first instance, we attempted to crystallize recombinant *Tc*KRS1 in the presence of either DMU371 or DMU759. However, despite multiple attempts in a wide range of crystallization conditions, high quality crystals could not be generated. Previous studies have demonstrated the propensity of KRS1 from *C. parvum* (*Cp*KRS1) to crystallize, producing crystals that diffract to high-resolution (21). Given that the ATP-binding pockets of the KRS1 enzymes of *T. cruzi* and *C. parvum* only differ by four amino acids, we synthesized a *T. cruzi/C. parvum* hybrid enzyme (*TcCp*KRS1) by mutating the four variable amino acids in the *Cp*KRS1 enzyme to the corresponding *T. cruzi* residues (I290L, A309S, M310V and I538L) (Fig. S4). This hybrid enzyme readily crystallized in the presence of lysine, with the resulting crystals then soaked in the presence of DMU371 or DMU759. *TcCp*KRS crystals soaked with DMU759 did not produce a liganded structure, however, those soaked with DMU371 diffracted to 1.6Å revealing *TcCp*KRS bound to inhibitor (table S7, Fig. 4A). DMU371 was bound within the ATP-binding pocket of the enzyme, with the quinazoline ring of the inhibitor in space usually occupied by the adenine ring of ATP, forming π-π stacking interactions with F307 (equivalent to F317 in *Tc*KRS1) and R523 (equivalent to R536 in *Tc*KRS1) (Fig. S5). The cyclopentyl moiety of DMU371 occupies a position similar to that adopted by the furan ring of ATP, in a pocket created by the lysine substrate S309 (equivalent to S319 in *Tc*KRS1) and surrounding amino acids and lined by three water molecules (Fig. 4A, B). We hypothesized that DMU759 occupied a similar binding position to DMU371. Given that this analog was slightly larger than DMU371, due to its cyclohexyl ring and bridging sulfur group, binding may have required a modest conformational change within the active site of KRS1 that was not possible in the rigid crystallized state. However, this modest change may have led to improved Van der Waals interactions between the inhibitor, lysine substrate and enzyme, explaining the superior potency of the DMU759 compound relative to DMU371 (Table 1). Based on our *TcCp*KRS1 liganded structure, mutation of S309 to leucine is likely to hinder the binding of DMU371 in a relatively minor way (Fig. 4C). In contrast, this mutation would likely cause a greater clash with the larger cyclohexyl group of DMU759, explaining the enhanced resistance seen with this analog compared to DMU371 (Table 1).

### *In vivo* efficacy of DMU759 in a mouse model of acute *T. cruzi* infection

Bioluminescent mouse models of infection have revolutionized our understanding of *T. cruzi* biology and Chagas disease pathogenesis (30, 31). We now recognize the dynamic nature of chronic infection with *T. cruzi*, the importance of the gut as a permissive niche for parasites, and the requirement to kill persister non-replicating parasite stages to achieve cure. In acknowledgment of the sub-optimal metabolic stability of both quinazoline compounds (Table S2), we opted to assess our most potent *in vitro* compound DMU759 in an acute rather than a chronic mouse model of *T. cruzi* infection. These initial studies were aimed at providing proof-of-concept that inhibitors of *Tc*KRS1 could reduce parasitemia *in vivo*.

**Fig. 5:**
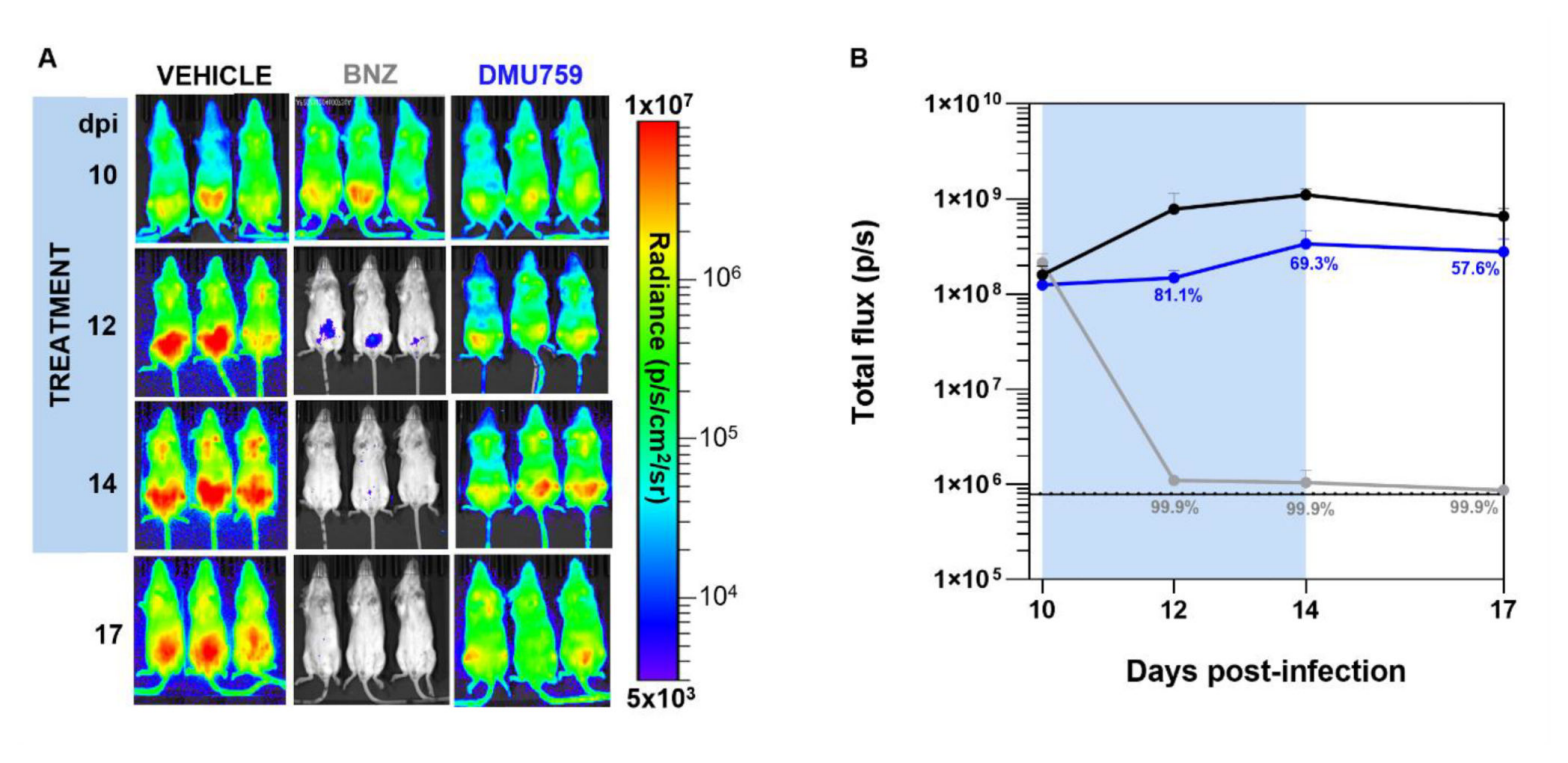
Assessment of DMU759 efficacy in a murine model of acute Chagas disease. (**A**) BALB/c mice infected with bioluminescent T. cruzi CL-Brener strain were treated for 10 days post-infection (dpi) for 5 days by oral gavage with 100 mg/kg benznidazole (BNZ) (n=3) once daily, or with DMU759 50 mg/kg (n=3) twice daily. Ventral images of each mouse are shown at various timepoints following infection. Heat maps are shown on a log_10_ scale and indicate bioluminescence intensity related to parasite burden (blue, low intensity; red, high intensity). The minimum and maximum radiances for the pseudocolor scale are shown. (**B**) Graph presents the mean bioluminescence (photons per second, p/s) determined by in vivo imaging of treated and untreated mice infected with T. cruzi. Treatment groups and dosing regimens are indicated. The black horizontal unbroken line indicates background bioluminescence total flux established from uninfected mice (n=3), with the dashed line indicating SD above the average.

To assess the *in vivo* efficacy of DMU759, BALB/c mice were acutely infected with *T. cruzi* CL Brener strain (10 days post-infection) and were treated orally for 5 consecutive days with 50 mg/kg DMU759 *BID* (*n*=3) and 100 mg/kg benznidazole *QD* (*n*=3) as a positive control (32). Parasite burden was monitored in treated and control mice via bioluminescence imaging. In mice treated with the standard of care treatment benznidazole, parasite burden was reduced close to the limit of detection, whereas treatment with DMU759 reduced parasite burden up to 81% (Fig. 5). Retrospective analysis of DMU759 concentration in blood following oral dosing of mice revealed that total blood concentrations comfortably exceeded EC_99_ (concentrations required to kill 99% of parasites) for up to 4 hours post dosing (Fig. S6). However, once these concentrations were corrected to account for DMU759 bound to plasma proteins (89% bound), it was confirmed that the free blood concentration of this compound did not exceed the established EC_99_ and only surpassed the EC_90_ for ~2 hours. In keeping with our earlier mouse liver microsome data (Table S2), DMU759 was also subject to rapid metabolism and blood concentrations rapidly declined after peaking one hour post dosing. Bearing in mind the pharmacokinetic limitations of DMU759 with regard to plasma protein binding and metabolic instability, the efficacy achieved with this compound *in vivo* suggests that pharmacokinetically optimized analogs from this series or others targeting KRS1 may have potential for effectively treating *T. cruzi* infection in this acute mouse model of Chagas disease.

## Discussion

In our current study, we used unbiased approaches to identify the molecular target of a new quinazoline series demonstrating promising phenotypic activity against a range of kinetoplastid parasites including *T. cruzi, T. brucei* and *L. donovani*. We identified the amino acyl tRNA synthetase KRS1 as the molecular target of these compounds. Focused studies in *T. cruzi* revealed that compounds from this series bound specifically within the ATP-binding pocket of KRS1. Of particular importance, *in vivo* studies in a murine model of acute Chagas disease provided proof-of-concept of the therapeutic potential of KRS1 inhibitors for treating *T. cruzi* infection. This efficacy was achieved despite sub-optimal metabolic stability of the test compound, DMU759. Future studies will focus on further developing the series to increase metabolic stability whilst retaining potency facilitated by our high-resolution liganded structure. This structure revealed a number of pockets close to the current inhibitor binding site (see Fig. 4B) that could be exploited to improve ligand binding. Metabolite identification studies will be vital to pinpoint the metabolic liabilities of this quinazoline series, however, we will also use two general strategies to improve stability namely to reduce LogD of the series and to look for the potential to achieve a scaffold hop. For instance, changing the cyclo-hexyl/pentyl ring would be a rapid route to reducing hydrophobicity within the series and changes at the 7-position of the quinazoline core have been shown to modulate phenotypic activity. H-bond interactions formed by the amino-quinazoline are well characterized thanks to our protein-ligand structure confirming the pyrimidine ring portion of this series will be difficult to replace, but this can be balanced by exploration of other 6,6 ring systems.

The fact that mutation of a single amino acid in the KRS1 active site could confer >20-fold resistance to the two lead compounds, DMU371 and DMU759, *in vitro* raises the possibility that this quinazoline series, or the target itself, may be associated with high drug resistance potential in the clinic. We have not yet assessed the relative fitness of parasites bearing KRS1 mutations *in vivo* and so cannot make any predictions regarding the likelihood of their transmission. Furthermore, the overwhelming majority of Chagas disease infections are zoonotic, with the animal reservoirs not previously having been exposed to drug treatment. This, together with the fact that parasite burden in patients with Chagas disease is low, greatly reduces the chances of acquired drug resistance emerging to any anti-chagasic treatment. However, the existence of naturally occurring resistance cannot be discounted. As required for any new chemical entity, our lead and candidate compounds will be screened to ensure they retain potency against clinical isolate panels. In addition, we will carefully monitor clinical isolate sequencing data to determine the frequency of mutations of concern within KRS1. Finally, we will look for appropriate partner compounds for our KRS1 inhibitors with a view to developing a combination therapy to further mitigate resistance risk.

Our understanding of the dynamics of *T. cruzi* infections has evolved considerably over the last decade. We have come to recognize the importance of persister forms of the parasite that are often less susceptible to drug treatment. We also recognize the requirement for drugs to kill all parasite forms to deliver sterile cure. *T. cruzi* persisters are considered transiently quiescent, however, we do not know the true metabolic status of these parasites. One could argue that as protein synthesis is likely downgraded in persisters that drugs targeting enzymes involved in protein synthesis, such as KRS1, may be less effective against slow growing or static forms of the parasite. The counter argument is that persister parasites will likely require some level of protein synthesis, particularly as they exit quiescence, thus KRS enzymes are viable drug targets. Notably, in other organisms, amino acyl tRNA synthetases can perform a broad range of essential, non-canonical functions beyond covalent binding of an amino acid to a corresponding tRNA (33, 34). Further work will be required to determine all of the functions performed by KRS1 in *T. cruzi* and to determine whether these functions are required for survival of persister forms of this parasite.

Our study has limitations. The current Target Product Profile guiding the development of anti-chagasic drugs stipulates that all future chemotherapies must be capable of treating both chronic and acute stages of the disease (35). To date, we have not been able to demonstrate that the quinazolines described in this study, or *Tc*KRS1 inhibitors in general, are capable of fulfilling these criteria. Having developed quinazolines with improved metabolic stability and reduced plasma protein binding, our goal will be to assess these compounds in chronic models of infection and in washout assays *in vitro* that will be used to examine the ability of compounds to deliver sterile cure *in vitro* (15, 36) and accurately predict efficacy *in vivo*.

In conclusion, our studies identified a promising chemical quinazoline series demonstrating partial efficacy in a murine model of acute Chagas disease. We chemically validated KRS1 as a promising drug target in *T. cruzi*. We hope that this research can be leveraged in the future to deliver much needed treatment options for this devastating infectious disease.

## Materials and Methods

### Study design

The objective of this study was to identify the molecular target of previously identified quinazoline compounds demonstrating promising *in vitro* activity against *T. cruzi* and related trypanosomatids. Our study aimed to provide target-based information that could be used to guide the development of quinazolines with improved pharmacokinetic properties while retaining potency. Multiple approaches were used to identify and validate the molecular target of two representative compounds from this series, DMU371 and DMU759. Approaches utilized included screening against our *T. brucei* genome-wide overexpression library, drug sensitivity assays against panels of transgenic parasite cell lines, isothermal protein profiling and recombinant *Tc*KRS1 inhibition assays. All biological assays were carried out in technical and biological replicate (≥2). Quantitative proteomics studies were carried out in technical replicate. Crystallographic studies were carried out to determine the binding orientation of our compounds in the active site of KRS1. Finally, the therapeutic potential of quinazolines targeting *Tc*KRS1 was assessed in a murine model of acute Chagas disease. Three mice, not predetermined using statistical methods, were randomly selected for inclusion in the study. A small cohort was used based on the high reproducibility of the bioluminescence mouse model and to adhere to animal welfare considerations. Investigators were not blinded to group allocation during experimental procedures or data analysis. Imaging data were collected as both optical images and quantitative bioluminescence, measured as total flux (photons/second), calculated from the sum of ventral and dorsal images. Quantification was performed manually by drawing individual regions of interest (ROIs) for each mouse, including naïve animals, to reduce potential selection bias and to increase the precision of area selection.

Animal work was performed under UK Home Office project license P9AEE04E4 and approved by the LSHTM Animal Welfare and Ethical Review Board. Procedures were performed in accordance with the UK Animals Scientific Procedures Act (1986).

### Cell lines and culture conditions

*T. cruzi* epimastigotes of the Silvio strain (MHOM/BR/78/Silvio; clone X10/7A) were maintained at 28°C in RPMI 1640 medium (Merck) supplemented with trypticase peptone (4.9 mg/ml), HEPES (20 mM), haemin (20 μg/ml), and foetal bovine serum (FBS, 10%), as previously described (29). *T. cruzi* metacyclic trypomastigotes were obtained and maintained, as previously described (29). Briefly, late-log epimastigotes were seeded at 10^6^ cells/ml in RTH/FBS and differentiated into trypomastigotes after 7 days at 28°C. Vero cells were infected with trypomastigotes for 12 hours in Dulbecco modified Eagle medium (DMEM) supplemented with 5% FBS, at 37°C in the presence of 5% CO_2_. Infected Vero cells were washed 3× with DMEM/FBS to remove extracellular parasites, and medium was replaced every two days until trypomastigotes re-emerged. Three rounds of infection were performed before trypomastigotes were harvested for drug sensitivity assays. For drug sensitivity assays carried out at the Swiss Tropical Institute, a *T. cruzi* cell line (Tulahuen C2C4) expressing the β-galactosidase gene *LacZ* was propagated in L6 rat skeletal myoblasts (ATCC CRL-1458) as previously described (37).

*T. brucei* bloodstream forms S427 (T7RPOL TETR NEO) and 2T1 were maintained at 37°C in HMI-11 medium (38) in the presence of 5% CO_2_. *T. b. rhodesiense* STIB 900 strain was maintained in HMI-9 medium supplemented with 15% heat-inactivated horse serum.

*L. donovani* (LdBOB) promastigotes derived from MHOM/SD/62/1S-CL2D were maintained at 28°C in modified M199 medium, as previously described (39).

Human HepG2 cells (ECACC 85011430) were cultivated at 37°C in minimal essential medium (MEM) supplemented with GlutaMax (1×, Thermo Fisher), 10% (v/v) fetal bovine serum (FBS), MEM non-essential amino acids (1×, Sigma) and in the presence of 5% CO_2,_ as previously described (29). Cells were passaged twice weekly through detachment of adherent cells with trypsin/EDTA (Sigma) and subsequent dilution into fresh medium.

Vero cells (African green monkey kidney epithelial cells, ECCAC 84113001) were maintained at 37°C, in 5% CO_2_, in DMEM supplemented with 10% FBS, as previously described (40). Cells were passaged twice weekly.

### Drug sensitivity assays

The relative potency of test compounds against *T. cruzi* epimastigotes (29), *T. b. brucei* bloodstream forms (41), *T. b. rhodesiense* bloodstream forms (42), *L. donovani* promastigotes (43) and Hep G2 cells (29) were established as previously described. The potency of test compounds on intracellular *T. cruzi* (Sylvio, X10/7A1) were determined in 96-well plates as previously described (29). Data were fitted to the two-parameter equation below, where *y* is % cell growth, *[I]* is the inhibitor concentration and *m* is the slope, using GraFit (Erithacus Software).

$$y=\frac{100}{1={\left(\frac{\left[I\right]}{EC_{50}}\right)}^{m}}$$

Drug sensitivity assays with *T. cruzi* (Tulahuen)-infected L6 rat skeletal myoblasts were carried out as previously described (37).

### Generation of a KRS1 overexpression construct and transgenic cell line

We began by assembling the genome of our in-house clone of *T. cruzi* X10/7, known as X10/7A1, using whole-genome sequencing (WGS) data derived from short reads via the SPAdes software (44). To identify the gene of interest (*TcKRS1*), we performed a homology search within the assembled genome using the CDS of TcSYL_0202360 (clone X10/1) as the query in the MMseqs software (45). Upon locating *TcKRS1*, we extracted a region encompassing 1000 base pairs upstream and downstream of the CDS. This region was used to generate the overexpression construct described below.

The *Tc*KRS1 CDS was synthesised by GeneArt (Thermo) and cloned into the *T. cruzi* expression vector pTREX (46) via EcoRI and XhoI restriction sites, generating pTREX-*Tc*KRS1. The accuracy of cloning was confirmed by Sanger sequencing using primers pTREX-Fw and pTREX-Rv (Table S8). Mid-log *T. cruzi* epimastigotes (2×10^7^) were transfected with 10 μg pTREX-*Tc*KRS1 using the Human T cell Nucleofector kit (Lonza) and Amaxa Nucleofector program U-033. Transfected cells were selected with G148 (250 μg/ml), and individual clones were generated by limiting dilution. Elevated *Tc*KRS1 expression in these transgenic clones, relative to wild type, was confirmed by quantitative proteomics.

The *Tb*KRS1 (Tb927.8.1600) ORF was synthesised by GeneArt (Invitrogen) and cloned into the tetracycline-inducible expression vector pRPa via HindIII and BamHI restriction sites (47), to generate pRPa-*Tb*KRS1 with the accuracy of cloning confirmed by Sanger sequencing using primers LBT-074 and LBT-075 (table S8). Mid-log *T. brucei* bloodstream trypanosomes (2×10^7^) were transfected with 10 μg pRPa-*Tb*KRS1 following digestion with AscI to linearise, using the Human T cell Nucleofector kit (Lonza) and Amaxa Nucleofector program X-001. Transfected parasites were seeded into a 96-well plate at a density of 3.2×10^3^ cells/ml under selection with hygromycin (2.5 μg/ml) and phleomycin (1 μg/ml) and single clones recovered. *Tb*KRS1 overexpression in harvested clones was evaluated by quantitative proteomics.

*Ld*KRS1 (LdBPK_150270.1) was amplified from *L. donovani* (LdBOB) gDNA by PCR using primers LBT-094 and LBT-095 (table S8) and cloned into the pIR1 expression vector via a BglII restriction site, generating pIR1-*Ld*KRS1. The accuracy of cloning was confirmed by Sanger sequencing with the sequencing primers pIR1-SAT-BglII-SeqF/R (table S8). Mid-log *L. donovani* promastigotes (10^7^) were transfected with linearised pIR1-*Ld*KRS1 (10 μg), following digestion of the plasmid with SwaI, using the Human T cell Nucleofector kit (Lonza) and Amaxa Nucleofector program V-033. Transfected cells were seeded in a 96-well plate at 100 cells/well under selection with nourseothricin (100 μg/ml) to generate clones. *Ld*KRS1 overexpression from resulting single cell clones was evaluated by quantitative proteomics.

### Generation of cell lines bearing KRS1 mutations

*T. cruzi* parasites bearing the S319L mutation in KRS1 were generated via CRISPR-free oligo-engineering (26). Wildtype epimastigotes (2×10^7^) were transfected with oligo LBT-148 (50 μg), as described above, and the resulting parasites were selected with DMU759 at 10× its established EC_50_ value. Parasites surviving DMU759 selection were cloned by limiting dilution, and four clones were selected for further evaluation. Genomic DNA was harvested from clones and the region of *Tc*KRS1 from nucleotide 827-1447 was PCR-amplified from gDNA recovered from the clones with primers LBT-165 and LBT-166. The resulting PCR products were Sanger sequenced using the primer KRS2-qPCR-Rev (table S8).

*T. brucei* KRS1 S323L mutants were also generated using CRISPR-free oligo-engineering. Bloodstream trypanosomes (2×10^7^) were transfected, as described previously, with oligo LBT-175 (50 μg). Transfected parasites were then selected with DMU759 at 10× its established EC_50_ value. The parasites surviving selection with DMU759 were then cloned by limiting dilution and four clones were selected for further evaluation. The *Tb*KRS1 ORF was amplified by PCR using primers JD081 and JD082 (table S8). The resulting PCR products were Sanger-sequenced with primer JD085.

*L. donovani* KRS1 S324L mutants were generated by CRISPR-Cas9 gene-editing in a WT background constitutively expressing Cas9 and T7 RNA polymerase, as previously described (48). In brief, a sgRNA template directing Cas9 cleavage of the endogenous KRS alleles was generated by PCR-elongation of primer LBT-115 with primer G00, as previously described (27). *L. donovani* promastigotes (2 × 10^7^) were transfected with repair oligo LBT-114 (50 μg). Transfected parasites were selected with DMU759 or DDD01510706 at 10× their respective EC_50_ values. Surviving parasites were cloned by limiting dilution, and four clones were selected for further evaluation. Genomic DNA was harvested from each clone and *Ld*KRS1 was PCR-amplified using primers LBT-094 and LBT-095. The resulting PCR products were Sanger-sequenced with primer LBT-085. All primers are shown in table S8.

### Quantitative proteomics analysis of wildtype and KRS1 overexpressing mutant

The relative protein abundance in the wildtype versus the overexpressing cell line was established as previously described (49), using 3×10^8^ parasites per sample. Here, proteins were identified by searching the protein sequence database containing *T. cruzi* Dm28c, *T. b. brucei* TREU927 or *L. donovani* BPK282A1 annotated proteins (downloaded from TriTrypDB 49 http://www.tritrypdb.org).

### *T. b. brucei* overexpression library screening and data analysis

DMU371 was screened against a tetracycline-inducible *T. b. brucei* overexpression library. The library was screened, and data processed, as previously described (19). In this instance, the library was selected with DMU371 at 2× its established EC_50_ value. Associated datasets have been deposited with the National Centre for Biotechnology Information Sequence Read Archive (NCBI SRA) under project code PRJNA1076710.

### Isothermal proteome profiling (iTPP)

*T. cruzi* epimastigote lysates were generated as previously described (29). Isothermal TPP assays were performed as previously described (25). In this instance, lysates were incubated with either DMU371 or DMU759 at concentrations equivalent to 10× their respective EC_50_ values or in the presence of vehicle (0.1% DMSO, drug-free control) for 30 min at RT and then submitted to a temperature shock for 3 min at 48°C. Data processing and analyses were performed as previously described (25), except proteins were identified via searches against the *T. cruzi* Dm28C proteome (tritrypdb.org, version 49).

### Cloning, expression and purification of recombinant KRS1

Cloning, expression and purification of recombinant *Hs*KRS has been described previously (21).

For *Tc*KRS1 (TcCLB.508971.30), synthetic DNA codon-optimized for expression in *E. coli* and encoding amino acid residues 54–580 of the *Tc*KRS1 protein was synthesized by Genscript and subcloned into a modified pET15b vector, with an *N*-terminal 6×His tag and a Tobacco Etch Virus (TEV) cleavage site. The resulting plasmid was transformed into *E. coli* BL21(DE3) (Stratagene) for expression. A 10-ml starter culture was grown at 37°C overnight, then used to inoculate 1 L of LB Autoinduction media that was grown for 48 h at 20°C. Cell pellets were resuspended in buffer containing 25 mM Tris, 500 mM NaCl, 20 mM imidazole, pH 8.5, and lysed using a continuous flow cell disrupter (Constant Systems). Recombinant *Tc*KRS1 was purified using Ni^2+^ affinity chromatography, the 6×His tag cleaved using TEV protease and the tag and protease removed by a second Ni^2+^ affinity chromatography step. The protein was further purified via size exclusion chromatography, where *Tc*KRS1 eluted with a mass consistent with the protein forming a dimer. Purified recombinant protein were concentrated to ~1 mg/ml in buffer (25 mM Tris, 150 mM NaCl, 10 % glycerol, pH 7.5) for assay.

Cloning, expression and purification of *TcCp*KRS1 was as described for *CpPf*KRS1 (25).

### *Tc*KRS1 inhibition assays using BIOMOL Green

The enzymatic activity of recombinant KRS was determined by monitoring the concentration of pyrophosphate released during the first step of the reaction catalyzed by recombinant *Tc*KRS1. The pyrophosphate formed was converted to two inorganic phosphate molecules using a pyrophosphatase enzyme and concentrations of the resulting phosphate were measured using the BIOMOL Green reagent (Enzo Life Sciences). All screening assays were performed in 384-well, clear, flat-bottom plates (Greiner) at room temperature (~23°C) in 50 μl reaction volumes.

To generate IC_50_ data for test compounds, 10-point inhibitor dose-response curves were prepared in 384-well assay plates using an ECHO 550 acoustic dispenser (Labcyte). Following preparation of the inhibitor curves, assays were carried out for recombinant KRS enzymes as follows: *Tc*KRS1 assay wells contained r*Tc*KRS1 assay buffer (100 mM Tris; pH 8, 140 mM NaCl, 30 mM KCl, 40 mM MgCl_2_, 0.01% (v/v) Brij and 1 mM DTT) plus 50 nM r*Tc*KRS1, 14 μM ATP (~2× *K_m_*), 400 μM L-lysine (~2× *K_m_*) and 0.5 U/ml pyrophosphatase; *Hs*KRS reactions contained *Hs*KRS assay buffer (30 mM Tris; pH 8, 140 mM NaCl, 30 mM KCl, 40 mM MgCl_2_, 0.01% (v/v) Brij and 1 mM DTT) plus 200 nM r*Hs*KRS, 3.5 μM ATP (~ 2× *K_m_*), 6 μM L-lysine (~3× *K_m_*) and 0.5 U/ml pyrophosphatase.

All assays were performed by adding 25 μl of assay buffer with enzyme to compound-containing assay wells before the reaction was initiated with the addition of a 25 μl substrate mixture containing L-lysine, ATP and pyrophosphatase. On all assay plates, 0% inhibition (DMSO) and 100% inhibition control wells were included (100% inhibition controls for the *Tc*KRS1 assay omitted lysine, while reference compound DDD01827593 was used at 100 μM for *Hs*KRS1 100% inhibition control wells). Following a 6–h reaction at room temperature, the assays were stopped by the addition of 50 μl BIOMOL Green. The BIOMOL Green signal was allowed to develop for 30 min before the absorbance of each well was read at 650 nm using a PheraStar plate reader (BMG). All liquid-dispensing steps were carried out using a Thermo Scientific WellMate dispenser (Matrix).

ActivityBase (IDBS, version 8.0.5.4) was used for data processing and analysis, with percentage inhibition values determined relative to 0% and 100% inhibition control wells. All IC_50_ curve fitting was undertaken using ActivityBase XE (version 7.7.1) from IDBS. Percentage inhibition curves for *Tc*KRS1 and human KRS, were fitted to a four-parameter logistic dose-response equation (XLfit model 203), with pre-fitting for all four parameters, using the equation below, where *A* is no inhibition, *B* is maximum inhibition, *C* is the Log_10_ (IC_50_), *D* is the Hill slope and *x* is the inhibitor concentration.

$$y=\frac{B-A}{1={\left(\frac{10^{\text{C}}}{x}\right)}^{D}}$$

### Crystallization of the*TcCp*KRS1 hybrid enzyme and structure determination

Crystals of *TcCp*KRS were grown in similar conditions to those previously used for crystallization of *Cp*KRS protein (21). Protein (30 mg/ml) in storage buffer (25 mM HEPES, 0.5 M NaCl, 5 % w/v glycerol, 2 mM TCEP, pH 7.0), was incubated with 5 mM lysine prior to setting up crystallization drops. Crystals were grown using vapor diffusion in hanging drops, with a reservoir containing 25% PEG 3350, 0.2 M lithium sulphate and 0.1 M Tris, pH 7.8. Crystallization drops consisted of 1 μl protein solution and 1 μl of reservoir. For soaking, crystals were transferred into drops consisting of 1 μl of reservoir and 1 μl storage buffer containing 10 mM compound. Crystals were harvested after one hour of soaking, cryoprotected using reservoir supplemented with 33% glycerol, and flash frozen in liquid nitrogen.

Data were collected at beamline I04 at Diamond Light Source, at a wavelength of 0.95373 Å. The data were integrated using the DIALS automated pipeline (50) and scaled and merged using Aimless (51). The structure was solved using the structure of *Cp*KRS (PDB: 5ELO, (21)) as the search model in Phaser (52). Manual model building was performed using Coot (53) and the structure refined using Refmac (54), incorporated into the CCP4 suite of software (55). The ligand dictionary was prepared using Acedrg (56) and model quality assessed using Molprobity (57). Data collection and refinement statistics are provided in table S7.

### Murine infections and bioluminescence imaging

Female BALB/c mice (aged 6-7 weeks), purchased from Charles River (UK), were maintained under specific pathogen-free conditions in individually ventilated cages, with a 12 h light/dark cycle. They had access to food and water *ad libitum*. Mice were infected by intraperitoneal injection (i.p) of 1x10^3^ bloodstream trypomastigotes derived from CB17 SCID mouse blood [27].

Compounds were prepared in 5% (v/v) dimethyl sulfoxide /95% HPMC suspension vehicle (0.5% (w/v) hydroxypropyl methylcellulose, 0.5% (v/v) benzyl alcohol, 0.4% (v/v) Tween 80 in Milli-Q water). Mice were treated as outlined in Fig. 5, with compounds administered by oral gavage. At different days after infection, mice were injected with d-luciferin (150 mg/kg) i.p., anaesthetized using 2.5% (v/v) gaseous isoflurane and imaged using an IVIS Spectrum (Revvity, Hopkinton, MA, USA) 5-10 min later. To estimate parasite burden, mice ventral and dorsal regions of interest were drawn using Living Image 4.7.3 to quantify bioluminescence expressed as total flux (photons/second; p/s). Regions of interest data from infected and not-infected mice were used as controls. Exposure times varied between 30 sec and 5 min, depending on the signal intensity.

### Determining DMU759 exposure in mice after oral dosing

DMU759 (50 mg/kg) was orally administered to BALB/c mice. The dose solution was prepared on the day of dosing, and the vehicle was 0.5% (w/v) hydroxypropyl methylcellulose and 0.4% (v/v) in Milli-Q H_2_O. Blood samples (10 μl) were collected from the tail vein of each animal at defined intervals then placed into 1.2 mL cryovials containing deionized water (90 μl) and stored at −80°C until analysis. Blood levels of DMU759 in mouse blood were determined by UPLC-MS/MS, as previously described (58).

### Plasma protein binding

The propensity of DMU759 to bind to plasma proteins was determined as previously described (21).

### Statistical analysis

EC_50_ curves for *in vitro* drug sensitivity assays were plotted using two-parameter non-linear regression. Where weighted means are reported, final values are generated from at least three biological replicates with each biological replicate comprised of two technical replicates. ActivityBase (IDBS, version 8.0.5.4) was used to analyze data generated from *Tc*KRS1 and human KRS enzyme inhibition assays. Percentage inhibition curves were fitted to a four-parameter logistic dose-response equation (XLfit model 203), with pre-fitting for all four parameters. IC_50_ values represent the geometric mean with 95% confidence intervals determined from at least three biological replicates (n = 3). The 95% confidence interval indicates the concentration range within which IC_50_ values are likely to fall.

## Supplementary Material

Supplementary Material

## Figures and Tables

**Table 1:** DMU759 and DMU371 EC_50_ values in wildtype and transgenic *T. cruzi* strains and human cell lines

Organism	Cell line	Parasite stage	DMU759 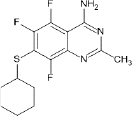		DMU371 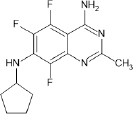	
			EC_50_ value, nM	Fold-shift (relative to WT)	EC_50_ value, nM	Fold-shift (relative to WT)
***T. cruzi*** (Sylvio X10/7A1 strain)	WT	Epimastigote	14 ± 0.3	-	63 ± 2.4	-
	KRS1^OE^		134 ± 3	10	624 ± 14	10
	KRS1^S319L^		565 ± 41	41	326 ± 26	5
	WT	Intracellular amastigote (in Vero cells)	16 ± 1	-	205 ± 24	-
	KRS1^OE^		121 ± 19	8	888 ± 117	4
	KRS1^S319L^		1540 ± 130	97	589 ± 57	3
***T. cruzi*** (Tulahuen strain)	WT	Intracellular amastigote (in L6 cells)	18 ± 7		233 ± 86	
**Human**	HepG2		16900 ± 5380		5820 ± 500	

EC_50_ values represent the weighted mean ± standard deviation of ≥2 biological replicates with each biological replicate comprised of ≥2 technical replicates. For *T. cruzi* (Tulahuen strain), EC_50_ values represent the mean +/- standard deviation of 4 biological replicates.

**Table 2:** Activity of quinazoline compounds in TcKRS1 and human KRS enzyme assays

Enzyme	IC_50_ values*, nM (95% CI)	
	DMU759	DMU371
***Tc*KRS**	15 (5 - 43)	185 (98 - 352)
**human** **KRS**	7170 (2860 - 18010)	9800 (5240 – 18340)

* IC_50_ values represent the mean with 95% confidence intervals determined from at least four biological replicates (n ≥ 4). The 95% confidence interval indicates the concentration range within which IC_50_ values are likely to fall.

## Data Availability

All data are available in the main text or supplementary materials. Mass spectrometry datasets have been deposited to the ProteomeXchange Consortium through the PRIDE (59) partner repository under the dataset identifier PXD050235. Genome-wide overexpression library datasets have been deposited in the National Centre for Biotechnology Information Sequence Read Archive (NCBI SRA) under project code PRJNA1076710. The coordinates and reflections file for crystallographic studies demonstrating DMU371 bound to *TcCp*KRS1 and lysine were submitted to the Protein Data Bank with the accession code 8S0V. DMU371 and DMU759 can be provided via an MTA by contacting ASB.

## One Sentence Summary

Quinazolines targeting lysyl tRNA synthase show partial efficacy in a mouse model of Chagas disease

## Accessible Summary

New drugs are required to treat Chagas disease, a deadly parasitic infection prevalent in Latin America. Here, Tulloch et al. investigate the mechanism of action of a new series of quinazoline compounds demonstrating potential against *Trypanosoma cruzi*, the causative agent of Chagas disease. Genetic, biochemical and proteomics approaches identify the molecular target of these compounds as lysyl-tRNA-synthetase-1. The lead compound from the series is partially efficacious in a model of acute Chagas disease thus identifying *T. cruzi* lysyl-tRNA-synthetase-1 as a viable drug target. However, this quinazoline series will require further development to become a future treatment for this neglected disease.
