# Supplementary material for: Anti-trypanosomal quinazolines targeting lysyl-tRNA synthetases show partial efficacy in a mouse model of acute Chagas disease
Source: Sci Transl Med. Author manuscript; Available in PMC 2025 Aug 1. (PMC7617978; doi:10.1126/scitranslmed.adu4564)
Supplement: Supplementary Material [file EMS207324-supplement-Supplementary_Material.pdf]

Supplementary Materials

Anti-trypanosomal quinazolines target lysyl-tRNA-synthetases and demonstrate partial efficacy in a model of acute Chagas infection

This file includes:

Supplementary materials and methods

Chemical synthesis

Tetrafluorinated intermediates (DMU371-I and DMU759-I)

4-(cyclopentylamino)-2,3,5,6-tetrafluorobenzonitrile (DMU371-I)

4-(cyclohexanethio)-2,3,5,6-tetrafluorobenzonitrile (DMU759-I)

Synthesis of DMU371 and DMU759

N7-Cyclopentyl-5,6,8-trifluoro-2-methylquinazoline-4,7-diamine (DMU371)

7-(Cyclohexylthio)-5,6,8-trifluoro-2-methylquinazolin-4-amine (DMU759)

DMPK assays

Metabolic stability

In vitro CYP inhibition

*In vitro h*ERG fluorescence polarization assay

Supplementary figures

Fig.S1. Schematic representation of the two-step aaRS reaction scheme (black arrows).

Fig.S2. Amino acid sequence similarity matrix (%) for a range of kinetoplastid, apicomplexan, bacterial and human KRS enzymes.

Fig.S3. Relative KRS1 protein levels in selected WT and transgenic *T. brucei*, *T. cruzi* and *L. donovani* cell lines.

Fig.S4. Alignment of *T. cruzi*, *C. parvum* and *P. falciparum* KRS1 genes.

Fig.S5. Crystal structure of the *TcCp*KRS active site with bound lysine and DMU371.

Fig. S6. Pharmacokinetic properties of DMU759.

Supplementary tables

Table S1: Collated EC_{50} values for WT and transgenic kinetoplastid cell lines.

Table S2: *In vitro* ADME profile of DMU371 and DMU759.

Table S3: Fragment hits from screening the *T. brucei* overexpression library with DMU371.

Table S4: Collated EC_{50} values for established KRS1 inhibitor DDD01510706 against WT and transgenic kinetoplastid cell lines.

Table S5: Top hits from isothermal TPP with DMU371.

Table S6: Top hits from isothermal TPP with DMU759.

Table S7: Statistics for the crystal structure of *TcCp*KRS1 with bound DMU371 and lysine.

Table S8: Primers used in this study.

Supplementary materials and methods

Chemical syntheses

Tetrafluorinated intermediates (DMU371-I and DMU759-I) - Pentafluorobenzonitrile (1.00 g, 5.18 mmol) and triethylamine (1.31 g, 12.95 mmol) were dissolved in THF followed by the addition of either cyclopentylamine or cyclohexanethiol (5.18 mmol). The mixture was stirred at RT for 12 h and subsequently quenched with water (100 ml). The crude product was extracted with ethyl acetate (3 × 50 ml). The combined organic extracts were dried over $MgSO_4$ and the solvent removed under vacuum. The crude product was recrystallised using EtOH to afford desired tetrafluorinated intermediates.

4-(cyclopentylamino)-2,3,5,6-tetrafluorobenzonitrile **(DMU371-I) -** Yellow solid: (0.94 g, 70%); mp 107 – 109 °C; ^{1}H-NMR (600 MHz, $CDCl_3$) δ 4.34 (s, 1H), 4.29 — 4.22 (m, 1H), 2.08 — 2.03 (m, 2H), 1.78 — 1.72 (m, 2H), 1.70 — 1.63 (m, 2H), 1.54 — 1.49 (m, 2H); ^{13}C-NMR (151 MHz, $CDCl_3$) δ 149.0 — 147.2 (m), 136.4 — 134.9 (m), 133.2 — 133.0 (m), 109.1 (t, J = 3.3 Hz), 78.7 (t, J = 17.7 Hz), 56.6 (t, J = 3.6 Hz), 34.8, 23.7; ^{19}F-NMR (565 MHz, $CDCl_3$) δ -135.5 (td, J = 17.6, 11.2 Hz, 2F), -159.9 (d, J = 14.1 Hz, 2F); HRMS: m/z found: 259.0862 $C_{12}H_{10}F_4N_2$ $[M+H]^+$ requires 259.0858.

4-(cyclohexanethio)-2,3,5,6-tetrafluorobenzonitrile **(DMU759-I) -** White solid: (1.39 g, 93%); mp 98 – 100 °C; ^{1}H-NMR (600 MHz, $CDCl_3$) δ 3.50 – 3.45 (m, 1H), 1.92 – 1.89 (m, 2H), 1.81 – 1.77 (m, 2H), 1.63 (td, J = 8.2, 4.0 Hz, 1H), 1.43 – 1.22 (m, 5H); ^{13}C-NMR (151 MHz, $CDCl_3$) δ 147.9 — 147.4 (m), 146.2 — 145.7 (m), 123.2 (t, J = 19.9 Hz), 107.6 (t, J = 3.6 Hz), 93.0 — 92.7 (m), 47.3, 33.6, 25.8, 25.4; ^{19}F-NMR (565 MHz, $CDCl_3$) δ -130.4 (q, J = 9.9 Hz, 2F), -132.6 (q, J = 11.3 Hz, 2F); HRMS: m/z found: 290.0627 $C_{13}H_{11}F_4NS$ $[M+H]^+$ requires 290.0627.

Synthesis of DMU371 and DMU759

The relevant tetrafluorinated intermediate (1.76 mmol), 1,8-diazabicyclo[5.4.0]undec-7-ene (DBU, 0.671 g, 4.41 mmol) and acetamidinium chloride (0.166 g, 1.76 mmol) were dissolved in DMF (3 ml) and stirred under reflux for 15 h. The reaction was quenched with water (100 ml) and the crude product extracted with DCM (dichloromethane, 3 × 15 ml). The combined extracts were dried over $MgSO_4$ and the solvent evaporated under vacuum. The product was purified using column chromatography (elution with DCM/MeOH).

N7-Cyclopentyl-5,6,8-trifluoro-2-methylquinazoline-4,7-diamine **(DMU371) -** White solid: (0.177 g, 34%); mp 231 – 233 °C; ^{1}H-NMR (600 MHz, $CDCl_3$) δ 5.81 (s, 2H), 4.34 (d, J = 7.6 Hz, 1H), 4.13 (t, J = 3.4 Hz, 1H), 2.57 (s, 3H), 2.09 — 2.03 (m, 2H), 1.78-1.71 (m, 2H), 1.68 — 1.63 (m, 2H), 1.53 (dt, J = 19.0, 6.5 Hz, 2H); ^{13}C-NMR (151 MHz, $CDCl_3$) δ 165.2, 158.6, 143.9 — 142.2 (m), 140.6 — 138.7 (m), 138.1 (d, J = 11.6 Hz), 137.3 (m), 130.5 (td, J = 12.1, 3.4 Hz), 93.6 (dd, J = 10.1, 2.9 Hz), 56.7, 34.7, 26.4, 23.8; ^{19}F-NMR (565 MHz, $CDCl_3$) δ -145.5 (m, 1F), -151.9 (m, 1F), -157.2 (dt, J = 20.7, 3.7 Hz, 1F); HRMS: m/z found: 297.1325 $C_{14}H_{15}F_3N4$ $[M+H]^+$ requires 297.1322.

7-(Cyclohexylthio)-5,6,8-trifluoro-2-methylquinazolin-4-amine **(DMU759) -** Yellow solid: 0.150 g (26%); mp 194 – 196 °C; ^{1}H-NMR (600 MHz, CDCl3) δ 6.04 (s, 2H), 3.44 (s, 1H), 2.63 (s, 3H), 1.92 (d, J = 10.6 Hz, 2H), 1.76 (s, 2H), 1.6 (1H, overlapped by H2O), 1.40 (d, J = 10.6 Hz, 2H), 1.31 — 1.22 (m, 3H); $^{13}C\{^1H,^{19}F\}$-NMR (125 MHz, $CDCl_3$) δ 165.6, 158.7, 153.6, 145.4, 142.1, 137.8, 117.9, 103.1, 47.0, 33.6, 26.4, 25.9, 25.5; ^{19}F-NMR (565 MHz, $CDCl_3$) δ -118.9 (d, J = 17.0 Hz, 1F), -134.4 (d, J = 25.4 Hz, 1F), -143.9 (t, J = 21.2 Hz, 1F); HRMS: m/z found: 328.1094 $C_{15}H_{16}F_3N_3S$ [M+H]+ requires 328.1090.

DMPK assays

Metabolic stability

The metabolic stability of key compounds was assessed using the protocol established by TCG Lifesciences (India). Briefly, mouse liver microsomes (MLM) were incubated with test compounds or controls (atenolol, propranolol, diclofenac and verapamil) at 1 μM. Reactions were initiated by the addition of NADPH and terminated by the addition of acetonitrile. Metabolism of test and control compounds at 5 different time-points was monitored via LC/MS/MS.

***In vitro* CYP inhibition**

Compounds were screened for CYP inhibition activity using the protocol established by TCG Lifesciences (India). The activity of five CYP450 isoforms: 1A2; 2C9; 2D6, 3A4; and 2C19 was monitored in the presence of test or control (miconazole) compounds at 10 μM. Reactions were terminated after 10 min (30 min for CYP2C19) by the addition of acetonitrile, centrifuged and analysed via LC-MS/MS.

***In vitro h*ERG fluorescence polarization assay**

Compounds were assessed for potential *h*ERG channel inhibition using the Predictor® hERG Fluorescence Polarization Assay Kit (Invitrogen), as per manufacturer's instructions. Compounds were screened with test compounds at concentrations ranging from 0.0003 – 30 μM. Fluorescence polarization was measured using EnVision plate reader fitted with polarized filters (excitation: 531 nm; emission: 595 nm). IC_{50} values were determined using GraphPad Prism software.

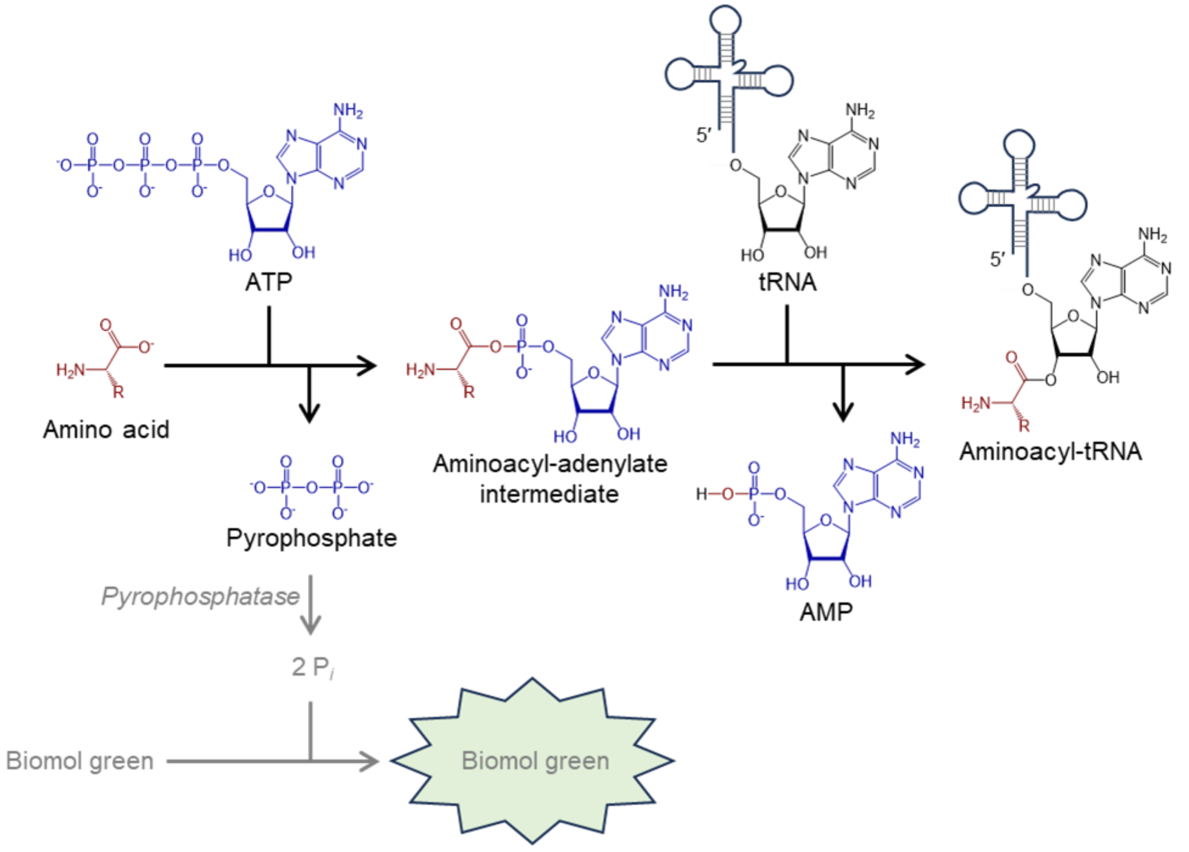

Fig. S1: Schematic representation of the two-step aaRS reaction scheme (black arrows). The coupled pyrophosphatase/BIOMOL® Green assay used to measure recombinant KRS1 enzymatic activity indicated in grey arrows.

	*Tc*KRS1	*Tb*KRS1	*Ld*KRS1	*Pf*KRS1	*Cp*KRS	*Hs*KRS1	*Tc*KRS2	*Tb*KRS2	*Ld*KRS2	*Mt*KRS
*Tc*KRS1	100	75	73	44	47	48	35	35	36	36
*Tb*KRS1	75	100	70	44	47	51	32	33	36	34
*Ld*KRS1	73	70	100	44	47	47	36	35	38	34
*Pf*KRS1	44	44	44	100	51	51	35	37	42	33
*Cp*KRS	47	47	47	51	100	53	35	37	40	34
*Hs*KRS1	48	51	47	51	53	100	34	36	41	35
*Tc*KRS2	35	32	36	35	35	34	100	68	64	34
*Tb*KRS2	35	33	35	37	37	36	68	100	65	34
*Ld*KRS2	36	36	38	42	40	41	64	65	100	37
*Mt*KRS	36	34	34	33	34	35	34	34	37	100

Fig. S2: Amino acid sequence similarity matrix (%) for a range of kinetoplastid, apicomplexan, bacterial and human KRS enzymes. The matrix was generated via sequence alignment using Clustal-Omega (version 1.2.4). Gene IDs as follows: *Tb*KRS1 (Tb927.8.1600); *Ld*KRS1 (LdBPK_150270), *Pf*KRS1 (XP_001350214.1), *Cp*KRS1 (XP_625825.1), *Hs*KRS1 (AAG30114.1), *Tc*KRS2 (TcKRS2.clone X10/7), *Tb*KRS2 (Tb927.6.1510), *Ld*KRS2 (LdBPK_300130), *Mt*KRS (WP_072520048.1).

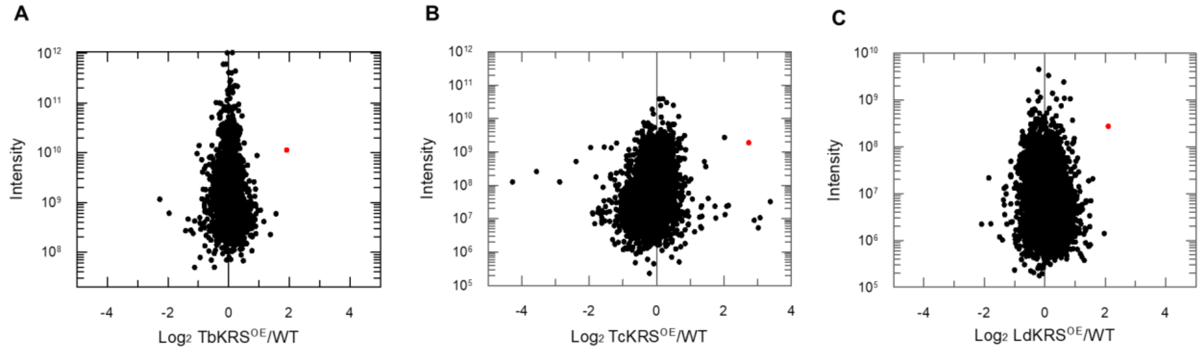

Fig. S3: Relative KRS1 protein levels in selected WT and transgenic *T. brucei*, *T. cruzi* and *L. donovani* cell lines. Protein levels (relative to WT) were determined by label-free quantitation. KRS1 relative expression in *T. brucei* (A), *T. cruzi* (B) and *L. donovani* (C) highlighted in red. Details of these analyses can be found in the Materials and Methods.

```
TcKRS1    MSS-------------------------TNETRAQIDD-LAAAIAQV--KKEKGAASEEC        32
PfKRS1    MTSKSFLLSFLKYKHVNTYIFEKSFSKILKNTKKHIDCHLKSCFVTMNEKKEHVLEGEKN       60
CpKRS1    MPCLSYILAALVETIIRLLFF-------------------FNYFVRMST-----------  30
          * .                            :. :

TcKRS1    RALVAKMTELRKQLPAKKVEKAPELSYFDTRLAMVKELGLLG-AAYPHKFDRQYTIPAFK   91
PfKRS1    KRVVNA----SKDKKKEEEGEVDPRLYFENRSKFIQDQKDKGINPYPHKFERTISIPEFI 116
CpKRS1    ----------EMNNQSQISDTLDSVHYTDNRYKMMECIKDAGRPFYPHKFKISMSLPAYA 80
                :   :        * :.*  :::     *   *****.   ::* :

TcKRS1    ARFAPQLSEKGQRVEEVVAIAGRIVNKRSSGSKLNFLTLQGDAETVQVISAISD--YVDD       149
PfKRS1    EKYKDL-GNGEHLEDTILNITGRIMRVSASGQKLRFFDLVGDGEKIQVLANYSFHNHEKG       175
CpKRS1    LKYGNV-ENGYIDKDTTLSLSGRVTSIRSSSSKLIFYDIFCEEQKVQIIANIMEHDISTG       139
          ::     :     :  : ::**:    :*..** *  :  : :..:*:::         .

TcKRS1    TFAAVHGRIRRGDIIGVKGVASLSKTGEFSMNAFEITLLSTCYHMLPDGWYGLSSIEQRF       209
PfKRS1    NFAECYDKIRRGDIVGIVGFPGKSKKGELSIFPKETILLSACLHMLPMK-YGLKDTEIRY       234
CpKRS1    EFSVSHSEIRRGDVVGFTGFPGKSKRGELSLFSKSVVLLSPCYHMLPTAISGLKDQEVRY       199
          *:  :..*****::*. *. .  ** **:*:   .  *** * ****     **.. * *:

TcKRS1    RQRYLDFIVNRENIQTFVTRSKVIRYIRNFFEDLDFLEVETPVLNQIAGGAAARPFITHH       269
PfKRS1    RQRYLDLLINESSRHTFVTRTKIINFLRNFLNERGFFEVETPMMNLIAGGANARPFITHH       294
CpKRS1    RQRYLDLMLNEESRKVFKLRSRAIKYIRNYFDRLGFLEVETPMLNMIYGGAAARPFITYH       259
          ******:::*... :.*  *:: *.::**:::  .*:*****::* * *** ******:*

TcKRS1    NELNQRMYLRIAPELYLKELVVGGMDRVYELGKQFRNEGIDLTHNPEFTSVEAYWAYADY 329
PfKRS1    NDLDLDLYLRIATELPLKMLIVGGIDKVYEIGKVFRNEGIDNTHNPEFTSCEFYWAYADY       354
CpKRS1    NELETQLYMRIAPELYLKQLIVGGLDKVYEIGKNFRNEGIDLTHNPEFTAMEFYMAYADY       319
          *:*:  :*:*** ** ** *:***:*:***:*** ******* *******: * * *****

TcKRS1    NDWMRTTEDLFYGLAMHIHGTPFVKYAPKDSEGNQLPEVVFNFNKPFKRLYIIPELEKRM       389
PfKRS1    NDLIKWSEDFFSQLVYHLFGTYKISYNKDGPENQP---IEIDFTPPYPKVSIVEEIEKVT 411
CpKRS1    YDLMDLTEELISGLVLEIHGSLKIPYHPDGPEGKC---IEIDFTTPWKRFSFVEEIESGL 376
          * :  :*:::  *. .:.*:  : *   .. *.:     : ::*. *: :. :: *:*.

TcKRS1    NVKFPTEFESDSSNAFLRELCSKHEVECIPPLTTARLLDALISHYLEPECQ-DPTFVCDH       448
PfKRS1    NTILEQPFDSNETIEKMINIIKEHKIELPNPPTAAKLLDQLASHFIENKYNDKPFFIVEH       471
CpKRS1    GEKLKRPLDSQENIDFMVEMCEKHEIELPHPRTAAKLLDKLAGHFVETKCT-NPSFIIDH       435
          . :   ::*:..   : ::  .:*::*    * *:*:*** * .*::* :    .* *: :*

TcKRS1    PRVMSPLAKWHRDDPQLTERFELFLNKKELCNAYTELNNPIVQREEFMKQLRNKEKGDDE 508
PfKRS1    PQIMSPLAKYHRTKPGLTERLEMFICGKEVLNAYTELNDPFKQKECFKLQQKDREKGDTE   531
CpKRS1    PQTMSPLAKWHREKPEMTERFELFVLGKELCNAYTELNEPLQQRKFFEQQADAKASGDVE 495
          *: ******:**  .* :***:*:*:  **: *******:*: *:: *  *    : .** *

TcKRS1    AMDIDEGFVQALEHALPPTGGWGLGIDRLVMFLTSQANIKEVLLFPAMKPETSSSLTYPP       568
PfKRS1    AAQLDSAFCTSLEYGLPPTGGLGLGIDRITMFLTNKNSIKDVILFPTMRPAN--------     583
CpKRS1    ACPIDETFCLALEHGLPPTGGWGLGIDRLIMFLADKNNIKEVILFPAMRNVKQNAQHSNQ       555
          *  :*. *  :**:.****** ******: ***:.:  .**:*:***:*:  .

TcKRS1    GTLLNGQGVPLL        580
PfKRS1    ------------        583
CpKRS1    H---SGN-----    559
```

Fig. S4: Alignment of *T. cruzi*, *C. parvum* and *P. falciparum* KRS1 genes. Sequences aligned using Clustal-Omega (version 1.2.4). *C. parvum* KRS1 residues mutated to their *T. cruzi* counterparts to generate the *TcCp*KRS1 hybrid enzyme are highlighted in yellow.

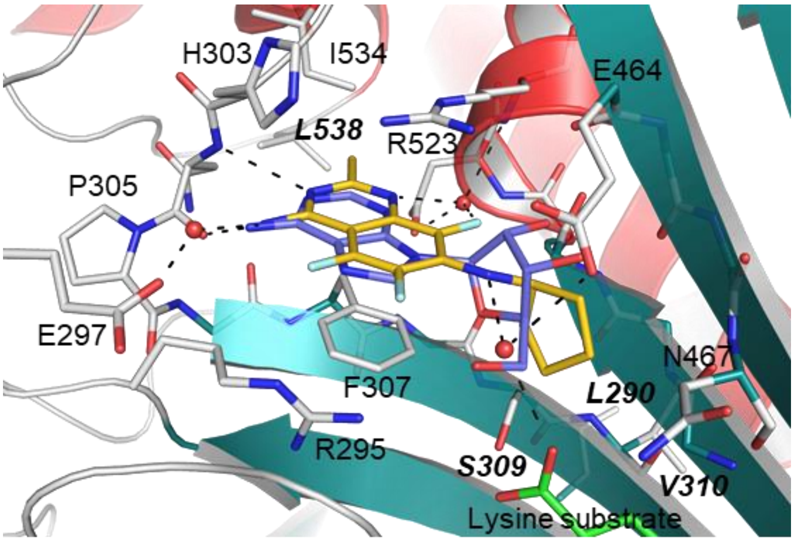

Fig. S5: **Crystal structure of the *TcCp*KRS active site with bound lysine and DMU371.** Lysine represented in green and DMU371 in gold. ATP (blue) binding position taken from *Hs*KRS (PDB ID: 3BJU) overlaid. Diphosphate is hidden for clarity. Key residues are labelled utilising *C. parvum* numbering, with residues mutated to *T. cruzi* counterparts in bold italics.

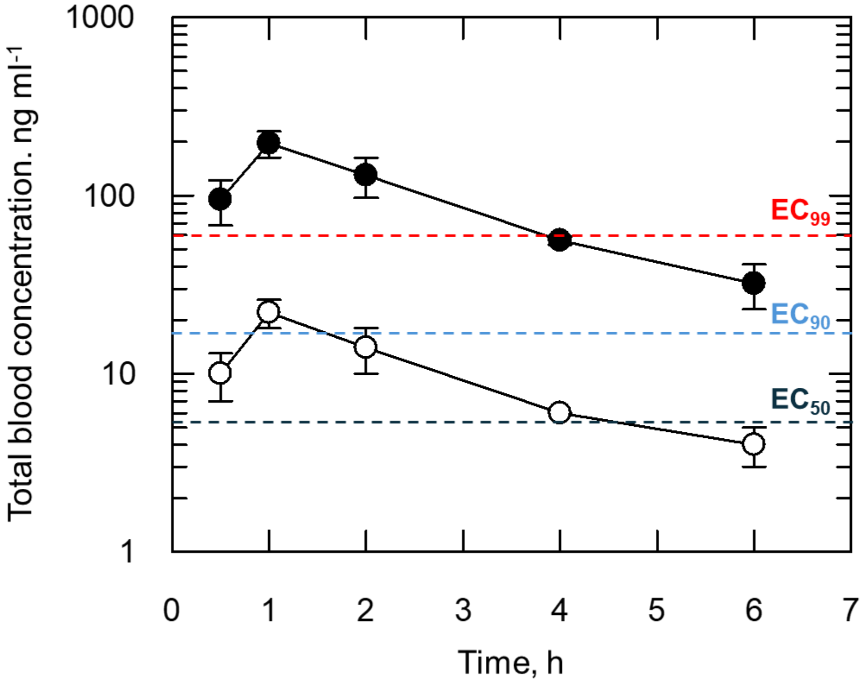

Fig. S6: Pharmacokinetic properties of DMU759. Total (black circles) and free DMU759 blood concentrations (open circles) following oral dosing (50 mg/kg). The EC_{99} (red), EC_{90}, (blue) and EC_{50} (dark blue) values of DMU759 for *T. cruzi* cultured in Vero cells are shown as dotted lines. Data represent the mean ± SD from three mice.

Table S1. Collated EC_{50} values for WT and transgenic kinetoplastid cell lines.

Organism	Cell line	Developmental stage	DMU759 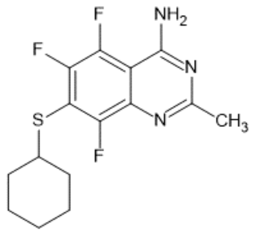		DMU371 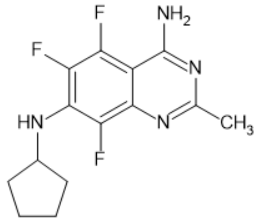	
			**EC_{50} value, nM**	**Fold-shift (relative to WT)**	**EC_{50} value, nM**	**Fold-shift (relative to WT)**
T. b. brucei	WT	BSF	112 ± 0.8	-	607 ± 9	-
	$KRS1^{OE}$		1060 ± 33	9	4250 ± 170	7
	$KRS1^{S319L}$		6480 ± 360	58	1870 ± 71	3
T. b. rhodesiense	WT		313 ± 183	-	1090 ± 420	-
L. donovani (LdBOB)	WT	Promastigote	115 ± 0.1	-	524 ± 9	-
	$KRS1^{OE}$		1470 ± 56	13	1860 ± 63	4
	$KRS1^{S324L}$		1320 ± 32	12	1740 ± 49	3
L. donovani (MHOM-ET-67/L82)	WT	Axenic amastigote	284 ± 20	-	5320 ± 2780	-

EC_{50} values represent the weighted mean ± standard deviation of at least ≥2 biological replicates with each biological replicate comprised of ≥2 technical replicates. For *T. brucei rhodesiense* and *L. donovani* MHOM-ET-67/L82, EC_{50} values represent the mean +/- standard deviation of 4 biological replicates.

Table S2: ***In vitro*** **ADME profile of DMU371 and DMU759.**

**Parameter**		**Compound**	
		**DMU371**	**DMU759**
**CYP inhibition (% at 10 µM**	**1A2**	74.8 ± 0.5	61.5 ± 0.9
	**2C9**	8.6 ± 0.6	7.9 ± 0.8
	**2D6**	6.3 ± 0.1	14.5 ± 2.5
	**3A4**	13.0 ± 3	0
	**2C19**	6.1 ± 1.3	12.8 ± 2.8
***h*ERG inhibition (IC_{50} value, µM)**		7.6 ± 1.3	12.2 ± 1.1
**Mouse liver microsome stability (µl/min/mg)**		>500	>500

Values represent the mean ± standard deviation from ≥2 biological replicates.

Table S3: Fragment hits from screening the *T. brucei* overexpression library with DMU371. Fragments containing genes with >400 RPKM are shown. Intact reading frames shown in blue with partial or incomplete gene fragments shown in grey.

Fragment	Gene ID	Gene name	RPKM	Reads	Fragment size (kbp)	Total Reads
**1**	Tb927.8.1600	lysyl-tRNA synthetase, putative	221733	1501190	4.3	**3,345,476**
	Tb927.8.1590	ubiquitin-protein ligase, putative	9235	455976		
**2**	Tb927.10.4050	serine palmitoyltransferase, putative	430	2754	4.7	**7,724**
	Tb927.10.4060	Ankyrin repeats (3 copies), putative	426	1802		

Table S4: Collated EC_{50} values for established KRS1 inhibitor DDD01510706 against WT and transgenic kinetoplastid cell lines.

Organism	Developmental stage	Cell line	DDD01510706	
			EC_{50}, µM	Fold-shift (relative to WT)
T. cruzi	Epimastigote	WT	1.4 ± 0.04	-
		$KRS1^{OE}$	13 ± 0.4	9
		$KRS1^{S319L}$	36 ± 4	25
T. b. brucei	BSF	WT	0.6 ± 0.01	-
		$KRS1^{OE}$	3 ± 0.1	5
		$KRS1^{S323L}$	33 ± 1	53
L. donovani	Promastigote	WT	0.7 ± 0.01	-
		$KRS1^{OE}$	8 ± 0.5	11
		$KRS1^{S324L}$	8 ± 0.3	12

EC_{50} values represent the weighted mean ± standard deviation of at least ≥2 biological replicates with each biological replicate comprised of ≥2 technical replicates.

Table S5. Top hits from isothermal TPP with DMU371.

Hit	Abundance Log$_2$ fold change		Peptides	Gene ID	Protein
	Rep 1	Rep 2			
1	2.50	2.97	22	C4B63_45g239	Lysyl-tRNA synthetase 1 (KRS1)
2	3.04	1.56	4	C4B63_42g593	Small nuclear ribonucleoprotein Sm-E
3	2.05	1.61	4	C4B63_49g169	Conserved hypothetical protein

Table S6. Top hits from isothermal TPP with DMU759. A total of 2365 proteins were detected by the presence of >2 peptides in two replicates. Those with a 1.5-$\log_2$ fold abundance change in both replicates are listed below.

Hit	Abundance $\log_2$ fold change Rep 1	Abundance $\log_2$ fold change Rep 2	Peptides	Gene ID	Protein
1	2.26	1.94	23	C4B63_45g239	Lysyl-tRNA synthetase 1 (KRS1)
2	-2.64	-3.79	9	C4B63_14g170	19S proteasome regulatory subunit
3	-2.04	-2.35	6	C4B63_3g1101	Mitochondrial processing peptidase, beta subunit
4	-2.27	-1.74	9	C4B63_116g3	P-type H^+-ATPase
5	-2.25	-1.75	5	C4B63_42g251	Conserved hypothetical protein
6	-1.59	-1.80	5	C4B63_6g2300	Ribosomal protein S26
7	-1.58	-2.80	8	C4B63_22g162	cysteine peptidase, Clan CA, family C2

Table S7: Statistics for the crystal structure of *TcCp*KRS1 with bound DMU371 and lysine.

Beamline	Diamond I04
Detector	DECTRIS EIGER2 XE 16M
Wavelength (Å)	0.95373
Space group	$P22_12_1$
Unit cell lengths (*a, b, c* Å)	73.14, 116.70, 142.88
Unit cell angles (α,β,γ °)	90, 90, 90
Resolution range (Å)	58.42 – 1.60 (1.64 – 1.60)
Total no. of reflections	2111816 (62276)
Total unique reflections	160925 (7506)
Redundancy	13.1 (8.3)
Completeness	99.9 (95.0)
R_{sym}	0.082 (1.212)
R_{pim}	0.033 (0.635)
<I>/<σ>	16.0 (1.6)
$CC_{1/2}$	0.999 (0.628)
R_{work} / R_{free}	18.43 (20.85)
B-factors	
Protein (A/B)	24.1 / 25.3
Ligand (A/B)	22.3 / 21.7
Lysine (A/B)	17.9 /18.9
Waters	29.9
R.m.s. deviations	
Bond lengths (Å)	0.0111
Bond angles (°)	1.664
Ramachandran favoured / allowed / disallowed (%, all chains)	98 / 2/ 0
PDB code	8s0v

Table S8. **Primers used in this study**.

Primer	Sequence (5'-3')
G00	AAAAGCACCGACTCGGTGCCACTTTTTCAAGTTGATAACGGACTAGCCTTATTTTAACTTGCTATTTCTAGCTCTAAAAC
JD081	TTAATTAAGCTTATGTCGGCTGTGGAGGAGCTCCGAGC
JD082	TGGGCAGGATCCTCAAAGAAGTGGCACCC
JD085	ACCTGCGAATTGCACCGG
KRS2-qPCR-Rev	TTGAGGGTCATTACGGTGCC
LBT 074	CAATGATAGAGTGGTACCC
LBT 075	TTACTCCGGGAACCTTTC
LBT 082	CTAGTGAGGCGTGCAAATCC
LBT 083	CGTTGACCCTTTTCCTGCAA
LBT 084	ACATCGACCAGTCCAAGTTCG
LBT 094	TTAATAAGATCTATGTCGTCCCTCGAAGAGCTCCGTAAGC
LBT 095	TTAATAAGATCTCTACAGCAGGGGAACACCCTGACC
LBT 114	TCGACCTGACCCACAACCCCGAATTTACGNNNTGCGAAGCGTACTGGGCATACATGGACTACC
LBT 115	GAAATTAATACGACTCACTATAGGCCCCGAATTCACAAGCTGCGGTTTTAGAGCTAGAAATAGC
LBT 148	ACCTCACACACAACCCGGAGTTTACCTTAGTAGAGGCGTACTGGGCCTACGCCGACT
LBT 165	TGTACCTGCGCATTGCTCCC
LBT 166	TGTACGCGTTGCAGAGCTCC
LBT 175	AGTCTGCGTACGCCCAATAACTCTCAACAAGTGTGAACTCCGGATTATGCGTGAGAT
pIR1-SAT-BglII-SeqF	TCATTGCTTCCTTCTGTTCCCCTCG
pIR1-SAT-BglII-SeqR	TGGTCGTAGAAATCAGCCAGTACAT
pTREX-F	CATTTTCACGCACGAAAGCG
pTREX-R	CTCGAGCCATTTACGAC
